# A new human *in vitro* model of cytotypic and testosterone-producing organoids derived from testicular tissue of transgender women

**DOI:** 10.1093/hropen/hoaf043

**Published:** 2025-08-20

**Authors:** Samuel Madureira Silva, Frédéric Chalmel, Andrea Errico, Katerina Papageorgiou, Guillaume Richer, Edith Chan Sock Peng, Antoine D Rolland, Kelly Tilleman, Guy T'Sjoen, Ilaria Dando, Tamara Vanhaecke, Ellen Goossens, Yoni Baert

**Affiliations:** Biology of the Testis (BITE) Laboratory, Genetics, Reproduction and Development (GRAD) Research Group, Vrije Universiteit Brussel, Brussels, Belgium; University of Rennes, Inserm, EHESP, Institut de Recherche en Santé, Environnement et Travail (Irset) UMR_S 1085, Rennes, France; Department of Neurosciences, Biomedicine and Movement Sciences, University of Verona, Verona, Italy; Biology of the Testis (BITE) Laboratory, Genetics, Reproduction and Development (GRAD) Research Group, Vrije Universiteit Brussel, Brussels, Belgium; In vitro Toxicology and Dermato-Cosmetology (IVTD) Research Group, Vrije Universiteit Brussel, Brussels, Belgium; Biology of the Testis (BITE) Laboratory, Genetics, Reproduction and Development (GRAD) Research Group, Vrije Universiteit Brussel, Brussels, Belgium; University of Rennes, Inserm, EHESP, Institut de Recherche en Santé, Environnement et Travail (Irset) UMR_S 1085, Rennes, France; University of Rennes, Inserm, EHESP, Institut de Recherche en Santé, Environnement et Travail (Irset) UMR_S 1085, Rennes, France; Department for Reproductive Medicine, Ghent University Hospital, Ghent, Belgium; Department of Endocrinology and Centre for Sexology and Gender, Ghent University Hospital, Ghent, Belgium; Department of Neurosciences, Biomedicine and Movement Sciences, University of Verona, Verona, Italy; In vitro Toxicology and Dermato-Cosmetology (IVTD) Research Group, Vrije Universiteit Brussel, Brussels, Belgium; Biology of the Testis (BITE) Laboratory, Genetics, Reproduction and Development (GRAD) Research Group, Vrije Universiteit Brussel, Brussels, Belgium; Biology of the Testis (BITE) Laboratory, Genetics, Reproduction and Development (GRAD) Research Group, Vrije Universiteit Brussel, Brussels, Belgium; In vitro Toxicology and Dermato-Cosmetology (IVTD) Research Group, Vrije Universiteit Brussel, Brussels, Belgium

**Keywords:** testicular organoids, testicular tissue of transgender women, new approach methodologies (NAMs), reproductive toxicology, endocrine disruption, *in vitro* testis model, testosterone production, 3D cell culture, human-based model, alternative to animal testing

## Abstract

**STUDY QUESTION:**

Can testicular tissue from trans women (trans tissue) be used to create human testicular organoids?

**SUMMARY ANSWER:**

Testosterone-producing and cytotypic human testicular organoids with bicompartmental architecture can be successfully generated from trans tissue.

**WHAT IS KNOWN ALREADY:**

Testicular organoids are a promising tool for studying testicular function and the effects of toxicants. Immature testicular cells are currently the most efficient at forming organoids that closely recapitulate seminiferous tubule-like architecture and functions. However, the scarcity of immature human testicular tissue limits its use in high-throughput applications. Conversely, trans tissue is abundantly available and characterized by an immature phenotype.

**STUDY DESIGN, SIZE, DURATION:**

Trans tissue-derived organoids (trans organoids) were histologically and androgenically compared to reference organoids derived from immature (prepubertal and pubertal) and adult cisgender testicular tissues. Additionally, long-term testosterone production and gonadotrophic stimulation were assessed in trans organoids. To evaluate their cytotypic and transcriptomic resemblance to reference testicular tissue stages, trans organoids were compared at the gene expression level to prepubertal, pubertal, and adult cisgender tissues, along with their tissue of origin.

**PARTICIPANTS/MATERIALS, SETTING, METHODS:**

Testicular tissue samples from transgender women, as well as from prepubertal, pubertal, and adult cisgender donors, were used to generate testicular organoids and to compare organoid formation efficiency and testosterone production according to tissue origin. These samples also served as references for transcriptomic comparisons with organoids derived from transgender women’s testicular tissue at Day 14 of culture. Testicular organoids were generated and cultured using 3D Petri Dish^®^ platforms. Histochemistry and immunofluorescence staining were employed to characterize cellular composition and spatial organization. Testosterone production in culture media was assessed using electrochemiluminescence immunoassays. RNA was extracted and sequenced from organoids derived from transgender women, as well as from tissue samples of all donor groups. Deconvolution and differential gene expression analyses were performed to compare the organoids with testicular tissues across all groups.

**MAIN RESULTS AND THE ROLE OF CHANCE:**

Trans organoids form compartmentalized, cytotypic *de novo* tissues similar to those from pubertal testicular tissue. Additionally, trans organoids exhibit significant testosterone production, sustain this function over extended culture periods, and respond to gonadotrophic stimulation. Deconvolved bulk RNAseq data indicate that cell population proportions within these organoids are close to those in prepubertal and pubertal testicular tissues. Gene expression clusters trans organoids alongside prepubertal and trans tissues. Functional analysis reveals that trans organoids share with prepubertal, pubertal, and trans tissues varied cellular processes. Factors such as the duration of hormone therapy, the expression of anti-Müllerian hormone—an immaturity marker—within the tubules, and the proportion of peritubular myoid cells in the donor tissue were found to predict the success of trans organoid formation.

**LARGE SCALE DATA:**

The bulk RNA-seq raw and preprocessed data are stored under restricted access in the Vrije Universiteit Brussel (VUB) Institutional Data Repository (VUB/IVTD/1/000001) due to participant privacy concerns. Access to the data will be considered by contacting Prof. Yoni Baert (yoni.baert@vub.be).

**LIMITATIONS, REASONS FOR CAUTION:**

Hormonal data from trans women donors were not acquired in a convenient manner for this study. Deconvolution data allow only cell proportions to be compared, not absolute numbers.

**WIDER IMPLICATIONS OF THE FINDINGS:**

This study highlights the potential of trans organoids as a novel and ethically sustainable human-based model for male reproductive health research, reproductive toxicology, and endocrine disruption studies. While trans tissue is a valuable replacement for immature tissue, further research should focus on optimizing organoid architecture, evaluating their utility in reprotoxicity testing, and promoting germ cell differentiation.

**STUDY FUNDING/COMPETING INTEREST(S):**

This study was conducted with financial support from the VUB Research Council (OZR4004) to S.M.S., the Scientific Research Foundation-Flanders (G026223N) and the Scientific Fund Willy Gepts to Y.B., the Strategic Research Program 89 from the VUB to E.G., and the Mireille Aerens Chair to T.V. The authors declare no conflict of interest.

WHAT DOES THIS MEAN FOR PATIENTS? Understanding how the testicles work is important for studying male fertility and how certain chemicals might affect it. Scientists often use small, lab-grown versions of organs—called organoids—to study these processes. However, making realistic testicular organoids has been difficult, especially because the best source of tissue (from young boys) is very limited. In this study, researchers discovered that testicular tissue from transgender women who have undergone gender-affirming surgery can be used to create testicular organoids in the lab. This tissue is usually discarded after surgery, but it turns out to be a valuable and ethical source for research. The organoids made from this tissue were able to produce testosterone and retain important testicular cells similar to those of developing testicles in early puberty. They also responded to hormone stimulation and could be kept alive in the lab for several weeks. This makes them a promising, more accessible, and human-relevant model for studying male reproductive health, testing the safety of chemicals, and possibly even supporting future fertility treatments.

## Introduction

It is estimated that 17.5% of the world’s population have experienced infertility in their life (World Health Organization, 2023). However, male infertility is often overlooked, while indeed the male factor contributes to, or is the cause of infertility in 50% of infertile couples (Hull *et al.*, 1985; Kumar and Singh, 2015). Although different possible causes contribute to this, it is known that the male reproductive system is a target of many substances that directly or indirectly impair fertility (Mortimer *et al.*, 2013). Moreover, fertility is associated with overall health and, thus, should not be just overcome by assisted reproduction techniques but rather by understanding the mechanisms responsible for the problems (Olea *et al.*, 2010).

Continuous exposure to environmental pollutants, many of which act as endocrine disruptors, leads to various male reproductive dysfunctions that may even originate as early as foetal development or childhood (Sharpe, 2006, 2010; Skakkebaek *et al.*, 2011). Many toxicants target gonadotrophin production directly, mainly in foetal life, or disturb steroidogenesis at the genomic or proteomic levels, leading to disturbed masculinization and infertility in adulthood (Mutoh *et al.*, 2006; Klinefelter *et al.*, 2012; Lymperi and Giwercman, 2018; Henriques *et al.*, 2019; Rodprasert *et al.*, 2021; Zhao *et al.*, 2022). Unfortunately, human studies are predominantly epidemiological and consequently circumstantial (Tingen *et al.*, 2004).

At present, both *in vitro* (OECD, 2020, 2022) and *in vivo* (OECD, 2018) assays are used for regulatory testing to evaluate oestrogenic and/or androgenic disruption. *In vitro* models offer some interesting advantages, such as their human origin and the expression of all enzymes needed for steroidogenesis, as in the case of the adenocarcinoma cell line (OECD, 2022). However, they are still far from mimicking the testicular environment. For reproductive toxicology, which includes testicular toxicity, the rat is the most commonly used *in vivo* model due to the extensive pharmacological knowledge and toxicological data available for this species (European Medicines Agency, 2018; OECD, 2018). Yet, studies performed in animal models are not easy to translate into relevant insights for human health, raise ethical concerns, and are expensive (Krewski *et al.*, 2010).

A culture system that models both testicular histology and functions (testosterone production and spermatogenesis) is an important and much sought-after tool in reproductive research. However, only mouse and rat testicular organ cultures lived up to this expectation (Sato *et al.*, 2011; Matsumura *et al.*, 2023)—and only if using certain strains (Portela *et al.*, 2019b)—despite many efforts with other mammals (Roulet *et al.*, 2006; de Michele *et al.*, 2018; Portela *et al.*, 2019a; Ibtisham *et al.*, 2023a, 2023b). Although organ cultures can maintain the testicular environment over relatively long periods (Komeya *et al.*, 2016), they are not as versatile as organoids. Organoids are miniaturized and simplified *in vitro* organs with similar histology and biological processes as the organ intended to mimic. Derived from cell suspensions of stem or differentiated cells that have self-organizing capabilities, they offer scalability and tailorability—very important features for a toxicological model. These properties allow large-scale testing by miniaturizing organoids and integrating them in a multi-organ-on-chip system, as demonstrated with testicular and liver organoids (Baert *et al.*, 2020). In addition, such a model also enables manipulation of cell composition (cell inclusion/exclusion) and cells (gene editing), making it a valuable tool for both basic and therapy-oriented reproductive research.

Human testicular organoids derived from prepubertal tissue were shown to be efficient in testosterone production (Cui *et al.*, 2024), which is one of the important functions of the testis. In contrast, when derived from adult tissue, organoids exhibit low testosterone levels throughout culture (Baert *et al.*, 2017, 2020). Testosterone production could also be detected in animal-derived organoid cultures; however, it is only particularly high when stimulated with a concentration of 4500 IU/l hCG (Edmonds and Woodruff, 2020; Cham *et al.*, 2024).

Despite the efforts made so far, human testicular organoids do not yet match the histological resemblance or germ cell differentiation standards of animal-derived testicular organoids. Only mouse and porcine organoids have shown a seminiferous-like epithelium surrounded by an interstitial-like compartment (Edmonds and Woodruff, 2020; Cham *et al.*, 2021; Richer *et al.*, 2021, 2024). However, immature testicular tissue is required to achieve such resemblance (Edmonds and Woodruff, 2020). Similarly, organoids derived from adult human tissue do not show any specific organization (Baert *et al.*, 2017), while prepubertal tissue shows the ability to form different cell compartments (Sakib *et al.*, 2019) or, in the best cases, seminiferous cord-like structures (Oliver *et al.*, 2021; Cui *et al.*, 2024), although not to the same level of histological resemblance as seen for mouse or porcine organoids. Regarding germ cell differentiation, claims of human *in vitro* spermatogenesis (Pendergraft *et al.*, 2017) are not sufficiently assessed to meet the standards suggested in the literature (Handel *et al.*, 2014; Lei *et al.*, 2021, 2023). In mouse organoids, spermatogenesis up to spermatid-like cells expressing postmeiotic cell-specific markers has been observed (Richer *et al.*, 2024; Stopel *et al.*, 2024), although these cells were still diploid (Richer *et al.*, 2024).

Furthermore, a high-throughput model based on human immature testicular tissue is not sustainable, and the generation of testicular cells from (induced) pluripotent stem cells is still at its beginnings, primarily limited to early testicular cell phenotypes (Gutiérrez *et al.*, 2018; Knarston *et al.*, 2020; Ishida *et al.*, 2021; Zhang *et al.*, 2021; Pryzhkova *et al.*, 2022). Recently, we showed that orchidectomized testicular tissue from trans women who have received gender-affirming hormone therapy (trans tissue) presents signs of rejuvenation and gene expression patterns similar to early pubertal tissue (Delgouffe *et al.*, 2025). The finding that Sertoli cells partially dedifferentiate, co-expressing androgen receptor (AR) and anti-Müllerian hormone (AMH), demonstrates potential for cell plasticity *in vitro*. Unlike immature testicular tissue, trans tissue is not scarce (van der Sluis *et al.*, 2020; The Brussels Times, 2022), as it is treated as medical waste material, which can make it an ethically sound source. Additionally, trans tissue can yield over a hundred million cells per testis. However, fibrosis was identified in the tissues (Delgouffe *et al.*, 2025), which might affect their usability. On the other hand, patients who stopped their hormone therapy for fertility reasons were able to restore spermatogenesis (de Nie *et al.*, 2023).

In this work, we explored the possibility of deriving human testicular organoids from trans tissue (trans organoids) using our most recent protocol for organoid formation (Richer *et al.*, 2024). These trans organoids were compared to human prepubertal, pubertal, and adult cisgender organoids (prepub, pub, and cis organoids) as references in terms of their histology and testosterone production. Additionally, we assessed the responsiveness of the steroidogenic system to gonadotrophic stimulation in trans organoids. To evaluate their level of cytotypic and transcriptomic resemblance to reference testicular tissue stages, trans organoids were compared at the gene expression level to prepubertal, pubertal, and adult cisgender tissues (prepub, pub, cis, and trans tissues), as well as to their tissue of origin.

## Materials and methods

### Gender-affirming hormone therapy

The trans women included in the study underwent a standardized diagnostic procedure and were at least 16 years old before starting gender-affirming hormone therapy (Dekker *et al.*, 2016), and at least 18 years old when orchidectomy took place. Treatment protocols were in accordance with the Standards of Care for the Health of Transgender and Gender Diverse People, Version 8 (Coleman *et al.*, 2022) and consisted of an oestrogen (Progynova^®^—median dose: 4.0 mg/day, dose range: 1.0–8.0 mg/day) along with an anti-androgen agent (Androcur^®^—median dose: 12.5 mg/day, dose range: 10–100 mg/day). Treatment duration varied from patient to patient, averaging 2.0 ± 1.7 years. To reduce the presumed risk of peri-operative thromboembolism, hormone therapy was stopped 2 weeks before orchidectomy in the five earliest donors, while in the six later donors, hormone therapy was continued until orchidectomy.

### Serum testosterone and oestrogen analysis of trans women

During the last pre-operative visit, a blood sample was collected to measure the participant’s testosterone and oestradiol levels using competitive chemiluminescent immunoassays. Testosterone was quantified with a E170 Modular system (Roche, Gen II, Mannheim, Germany) with a limit of quantification (LOQ) of 10 ng/dl (0.4 nmol/l) and an interassay CV of 2.6%, while oestradiol was measured using a E170 Modular system (Roche, Gen III) with a LOQ of 25 pg/ml and an interassay CV of 3.2%.

### Tissue sampling and cryopreservation

Cis tissue was obtained from men (n = 5) who underwent orchidectomy or vasectomy reversal in the Universitair Ziekenhuis (UZ) Brussel. Pub (n = 3) and prepub (n = 3) tissues were obtained from patients who underwent a testicular biopsy in the UZ Brussel in the context of fertility preservation and donated part of the biopsied tissue for research. Tissues were characterized as prepubertal, pubertal, or adult based on the age of the donors and seminiferous epithelium maturity (see Supplementary Table S1 for the age and histological characterisation of the donors). Prepubertal tissues were obtained from donors younger than 12 years old, showing no seminiferous lumen or spermatogenesis. Pubertal tissues were from donors between 12 and 16 years old, showing initiated spermatogenesis with maturing seminiferous epithelium but no spermatozoa. Adult tissues came from donors older than 16 years old, with spermatozoa present. Regarding the collection of trans tissue (n = 11; median age: 27 years, range: 18–53), the tunica albuginea and, if applicable, the rete testes were removed, and the testes were processed in pieces of ∼50 mm^3^. Tissue pieces were collected in Dulbecco’s modified essential medium/F12 (DMEM/F12; 11320-033, Gibco, Grand Island, NY, USA) and were pseudonymized by UZ Gent and transported to Vrije Universiteit Brussel (VUB) on ice. At VUB, tissue pieces were sectioned into smaller fragments and cryopreserved by slow freezing, as described before (Baert *et al.*, 2013). Prepub, pub, and cis tissues were cryopreserved in the same way. Briefly, tissue pieces (1–8 mm^3^) were equilibrated for 10 min in cryomedium consisting of 1.5 M dimethylsulphoxide (D2650, Sigma-Aldrich, Overijse, Belgium), 0.15 M sucrose (D21881000, PanReac AppliChem, Darmstadt, Germany), and 10 mg/ml (10%) human serum albumin (10064, Vitrolife, Londerzeel, Belgium) diluted in DMEM/F12 on ice. Before being stored in liquid nitrogen, the cryovials were placed in an isopropyl alcohol container (Mr Frosty, VWR, Philadelphia, PA, USA) for 24 h at −80°C. The collection and use of all tissues were approved by the Committee for Medical Ethics of the UZ Brussel—VUB (2016/036 and 2022/161) and UZ Gent (2014/1175 and 2014/1175-AM01). Patients or their parents gave written informed consent to donate testicular tissue to research.

### Tissue thawing and digestion

Cryopreserved testicular tissue was thawed by holding cryovials for 2 min in a 37°C water bath. The tissue pieces were washed twice for 5 min to remove the cryoprotectant: the first wash was performed in DMEM/F12 containing 10% (v/v) foetal bovine serum (FBS, 10500-056, Thermo Fisher, Seneffe, Belgium), and the second only in DMEM/F12. Single-cell suspensions were obtained by mechanical disruption and enzymatic digestion of the tissue pieces in DMEM/F12 containing 0.5 mg/ml DNAse (10104159001, Roche, Penzberg, Germany), 0.5 mg/ml hyaluronidase (37326-33-3, Sigma-Aldrich, Saint Louis, MO, USA), and 1 mg/ml collagenase Ia (05349907103, Roche) (Baert *et al.*, 2019). First, the tissue was mechanically disrupted using a 5 or 10 ml serological pipette, followed by a 15-min incubation at 37°C on a shaking plate (150 rpm). If digestion was incomplete after this period, subsequent mechanical disruption and 5-min incubation periods were added until digestion was fully achieved. After digestion, a 40 μm cell strainer (35234, BD Falcon, Leuven, Belgium) was used to remove remaining cell aggregates. Viable cell concentrations were determined using the 0.4% trypan blue (15250061, Thermo Fisher, Grand Island, NY, USA) exclusion test in a Neubauer chamber (718605, Blaubrand, Brand, Wertheim, Germany) observed under an inverted phase-contrast microscope (CKX53, Olympus, Hamburg, Germany). Only viable cells were considered for the final cell seeding densities.

### Testicular organoid culture

The protocol for generating human testicular organoids is similar to that used for the generation of our latest published mouse testicular organoids (Richer *et al.*, 2024), which relies on the self-assembly and reorganization potential of single testicular cells in a matrix-free scaffold. Cell suspensions were seeded in 3D Petri Dishes^®^ (3DPDs) made of 2% (w/v) agarose (16500100, Thermo Fisher, Carlsbad, CA, USA) gelled in moulds from MicroTissues^®^ (35 microwells, diameter 800 μm, depth 800 μm; Z764051-6EA, Sigma Aldrich, Cincinnati, OH, USA) at a density of 500 000 cells/3DPD, i.e. ∼14 000 cells/microwell. After casting, the 3DPDs were equilibrated at least twice for 15 min in the final desired medium and kept in 24-well plates during the culture. Medium refreshments of 300 μl were done every 2–3 days. The medium used for the cultivation of the organoids—referred to as complete StemPro (cSP) from here on—was chosen based on our previous findings describing it as optimal for testicular cell reorganization into organoids (Richer *et al.*, 2024). In short, the main components of cSP are StemPro™-34 serum-free medium (10639011, Gibco, Carlsbad, CA, USA) supplemented with 10% (v/v) KnockOut™ Serum Replacement (KSR; 10828010, Thermo Fisher, Grand Island, NY, USA), 1% (v/v) antibiotic antimycotic solution (100×) (A5955, Sigma-Aldrich, Hamburg, Germany), 20 ng/ml epidermal growth factor (PHGC311L, Gibco, Paisley, UK), 10 ng/ml fibroblast growth factor 2 (PMG0034, Gibco, Paisley, UK), 10 ng/ml glial cell line-derived neurotrophic factor (PHC7045, Gibco, Paisley, UK), 30 ng/ml β-oestradiol (E2758, Sigma-Aldrich, St Louis, MO, USA), 60 ng/ml progesterone (P8783, Sigma-Aldrich, St Louis, MO, USA), and 2.5% (v/v) StemPro supplement (10640-019, Gibco, Carlsbad, CA, USA). For the full composition, see Supplementary Table S2. For the serum-free conditions (cSPsf), KSR was left out from the medium composition. In experiments including stimulation of steroidogenesis, hCG (CG5-1VL, Sigma-Aldrich, St Louis, MO, USA) was added to the medium in concentrations of 1, 50, 100, and 4500 IU/l. Cultures were kept for 14 days, except for those with hCG conditions, which were kept for 26 or 63 days in 5% CO_2_ at 35°C. Additionally, to test the maintenance of the organoids over 63 days, one trans organoid culture was kept for 100 days. The reorganization of the testicular cells into organoids was followed up at every medium change under a brightfield microscope (CKX53, Olympus) throughout the whole culture period.

### Histochemistry and immunofluorescence staining and analyses

At the end of the 14-day cultures (and long-term 63-/100-day culture), organoids were enclosed in the 3DPD in which they were cultured by adding 2% (w/v) agarose on top. Enclosed organoids and tissue samples were fixed in acidified alcoholic formalin (PFAFA0060AF59001, Labonord, Saint-Apollinaire, France), dehydrated, and embedded in paraffin. Paraffin blocks were sliced into 5 μm serial sections with a sliding microtome (SM2010R, Leica, Nussloch, Germany). Sections were placed on SuperFrost^®^ Plus slides (631-9483, VWR, Braunschweig, Germany) and dried overnight in an oven at 37°C. Each section contained at least 20 of the 35 organoids per 3DPD. The sections were deparaffinized in xylene and rehydrated. After washing in phosphate-buffered saline (PBS; 70011051, ThermoFisher, Paisley, UK), the sections were stained with haematoxylin and periodic acid–Schiff (101646, Sigma-Aldrich, Darmstadt, Germany) to perform gross morphological evaluations of the tissues and organoids. Sections of organoids and tissue samples were also fluorescently stained. All immunofluorescent stainings (except the double-staining for SOX9/WT1) were performed with the same protocol. Shortly after washing in PBS, antigen retrieval was achieved through incubation of the slides at 121°C for 15 min in a Tris–EDTA buffer (pH 9). After a blocking step with 5% normal donkey serum (017-000-121; Jackson ImmunoResearch, West Grove, PA, USA), the sections were incubated with primary antibodies overnight in a humidified chamber at 4°C. After washing in PBS, the sections were incubated in a humidified chamber for 1 h with the secondary antibodies and Hoechst (1:2000; H3570, Life Technologies, Waltham, MA, USA) and mounted using ProlongTM Gold antifade reagent (P36934, Invitrogen, Waltham, MA, USA). To analyse the ratio of SOX9^+^/WT1^+^ cells, trans tissue slides were used. After washing in PBS, antigen retrieval was achieved through incubation of the slides at 121°C for 15 min in a Tris–EDTA buffer (pH 9). After a blocking step with 3% H_2_O_2_ in methanol, the sections were blocked with 5% normal goat serum (B304, Tebu-bio, Boechout, Belgium). The sections were incubated with the primary antibody for WT1 overnight in a humidified chamber at 4°C. After washing in PBS, the sections were incubated in a humidified chamber for 1 h with the horseradish peroxidase (HRP)-conjugated secondary antibody. The first colour was developed with cyanine 3 (NEL744001KT, Akoya Biosciences, Marlborough, MA, USA) incubation for 30–60 s. Second antigen retrieval was done in Tris–EDTA buffer (pH 9) for 60 min at 95°C in a water bath. The sections were blocked in 5% normal goat serum and then again incubated overnight in a humidified chamber at 4°C but this time with the primary antibody for SOX9. After washing in PBS, the sections were incubated in a humidified chamber for 1 h with the secondary antibody and Hoechst (1:2000) and mounted using ProlongTM Gold antifade reagent. The details of all antibodies used can be found in Supplementary Table S3. Slides were analysed using a brightfield/fluorescence microscope (CKX53, Olympus) and Toupview software (Version: x64, 4.11.19728.20211022, ToupTek, Hangzhou, China).

### Areas and cell counting

The percentage of the area occupied by seminiferous tubules in tissue sections was determined by measuring the areas of the tubules, including the tubular wall, over the total area analysed. The SOX9^+^/WT1^+^ ratio was determined by counting the number of SOX9^+^ and WT1^+^ Sertoli cells in the same tubules. The AMH^+^/AR^+^ ratio was determined by counting the number of AMH^+^ and AR^+^ tubules in a tissue section. Tissue and tubular area calculations as well as cell and tubule counting were performed using Toupview software.

### Testosterone analysis of culture media

Testosterone concentrations were assessed using the Elecsys Testosterone II competitive immunoassay (ms_05200067190V9.0, Cobas, Mannheim, Germany) on a Cobas 8000 bioanalyzer (Roche Diagnostics, Rotkreuz, Switzerland). Culture medium from all conditions was collected in Hitachi cups at the following time points: Days 2, 7, and 14 for all experiments, as well as Days 21, 28, 35, 49, and 63 for the long-term testosterone production experiment, or Days 21 and 26 for the cSP vs cSPsf experiment. Before analysis, samples underwent centrifugation to eliminate any precipitates. The assay employs a competitive test principle with a high-affinity monoclonal antibody specific to testosterone. In brief, 20 μl of the sample was incubated with a biotinylated monoclonal testosterone-specific antibody. Streptavidin-coated microparticles and a testosterone derivative labelled with a ruthenium complex were introduced to bind the formed complex to the solid phase via biotin–streptavidin interaction. After transferring the reaction mixture to wells, microparticles were magnetically captured onto the electrode surface, and unbound substances were eliminated by washing with ProCell/ProCell M (04880340190, Roche Diagnostics). Hormone concentrations were quantified using electrochemiluminescence, detected by a photomultiplier with a detection limit of 0.025 ng/ml. Concentrations were determined through instrument-specific calibration curves generated via two-point calibration and a master curve provided by the reagent barcode. The reported values were calculated by subtracting the reading from negative fresh-medium controls from the samples’ testosterone concentrations.

### Statistical analyses related to trans organoid architecture and testosterone production

Statistical analyses were performed using GraphPad Prism (version 10.4, GraphPad Software, Boston, MA, USA). The statistical tests used to analyse the data are specified in the legend of the graphical representations. Statistical significance was set at *P *< 0.05.

### RNA extraction and RNA sequencing

For gene expression analysis, cryopreserved testicular tissue from the same donors, except for one of the trans women, and organoids derived from the tissue donated by the same trans women were used. Cryopreserved testicular tissue was thawed as described in the ‘tissue thawing and digestion’ section. Per donor, 35–70 organoids were collected after 14 days in culture and used fresh. RNA extraction was performed with the QIAGEN RNeasy Micro Kit (74004, QIAGEN, Hilden, Germany). RNA concentration was assessed by using the NanoDrop^®^ ND-1000 UV-Vis Spectrophotometer (Thermo Fisher Scientific, Rodano, Italy). Using the DNF-472 High Sensitivity RNA Analysis kit (DNF-472-0500, Agilent Technologies, Santa Clara, CA, USA), the quality of the RNA samples was assessed on the AATI fragment analyser (Agilent Technologies). RNA libraries were created from 150 ng of total RNA using the KAPA RNA HyperPrep kit with RiboErase HMR (08098140702, Roche Diagnostics), according to the manufacturer’s instructions. In summary, following ribodepletion and DNase digestion, RNA was fragmented to average sizes of 200–300 bp by incubating the samples for 6 min at 94°C. After first-strand synthesis, second-strand synthesis, and adapter ligation, the libraries were amplified using 12 PCR cycles. Using the DNF-474 High Sensitivity NGS Fragment Analysis kit (DNF-474-0500, Agilent Technologies), final libraries were qualified on the AATI fragment analyser and quantified on the Qubit 2.0 with the Qubit dsDNA HS Assay Kit (Q32854, Life Technologies, Paisley, UK). Using the NovaSeq 6000 S4 Reagent Kit (200 cycles; 20028313, Illumina, San Diego, CA, USA), 25 million pairs of 100-base-long reads (through paired-end sequencing) were generated per sample on the NovaSeq 6000 system (Illumina). For this, 1.9 nM libraries were denatured according to the manufacturer’s instructions. After demultiplexing and an adaptor/quality trimming step, the raw reads were mapped against the human genome (hg19) using Spliced Transcripts Alignment to a Reference—STAR (Dobin *et al.*, 2013) and then translated into a quantitative measure of gene expression with the tool HTSeq (Anders *et al.*, 2015).

### Deconvolution analysis

To investigate the cellular composition of the samples, a cellular deconvolution approach using the MuSiC tool was employed (Wang *et al.*, 2019). This analysis integrated single-cell RNA-seq data from the developing human gonad meta-atlas, accessible through the ReproGenomics Viewer (RGV) database (Darde *et al.*, 2019). The meta-atlas aggregates data from 212 human gonadal samples spanning embryonic stages to adulthood, derived from 13 landmark studies, and includes a total of 714 432 cells. For our analysis, we specifically selected 331 712 cells from 102 distinct testis samples within this dataset. Each bulk sample was deconvolved to estimate the proportion of nineteen broad individual cell types (foetal germ cells, spermatogonia, spermatocytes, spermatids, foetal and adult Sertoli cells, rete testis cells, gonadal progenitor cells, gonadal coelomic epithelial cells, gonadal mesenchymal cells, undifferentiated supporting cells, foetal and adult Leydig cells, peritubular myoid cells, endothelial cells, embryonic erythrocytes, definitive erythrocytes, perivascular cells, and immune cells) described in the single-cell developmental meta-atlas of human gonads (Supplementary Fig. S1). Count matrices of single-cell RNA-seq data and bulk RNA-seq data were used as input data. To assist the deconvolution algorithm with cell-type-specific genes, the single-cell RNA-seq matrix was reduced to only variable genes. The proportions of the cell types were estimated by summing the predicted proportions of their associated cell clusters. Statistical comparisons between trans bicompartmental organoids (n = 6) and their original trans tissues (n = 6), along with the other tissue groups, were conducted using the Student’s *t*-test implemented in R (v4.3.0, Vienna, Austria). A subsequent analysis was performed, including all trans organoids and their tissues of origin. The cell proportions in the ‘bicompartmental’ and ‘irregular’ organoids and tissue groups were compared using Mann–Whitney *U*-tests in GraphPad Prism.

### Differential gene expression analysis

The UMI matrix was normalized with the regularized log (rlog) transformation package from DeSeq2 (Love *et al.*, 2014). Differentially expressed genes (DEGs) were identified based on the following statistical comparisons: (i) prepub vs pub tissue; (ii) prepub vs cis tissue; (iii) prepub vs trans tissue; (iv) prepub tissue vs trans organoids; (v) pub vs cis tissue; (vi) pub vs trans tissue; (vii) pub tissue vs trans organoids; (viii) cis vs trans tissue; (ix) cis tissue vs trans organoids; and, (x) trans tissue vs trans organoids. The AMEN suite was used to identify DEGs (Chalmel and Primig, 2008). A statistical comparison between the different types of testicular tissue was made to identify DEGs. In short, genes that were more expressed than the background cutoff (overall median of rlog-transformed UMI dataset, 2.03) and that exceeded 2.0-fold change were used for further analysis. Significant DEGs were identified by using the empirical Bayes moderated *t*-statistics implemented into the LIMMA package (Smyth, 2004; Ritchie *et al.*, 2015) with an adjusted *F*-value estimated using the Benjamini and Hochberg False Discovery rate approach (*P* ≤ 0.05) (Benjamini and Hochberg, 1995). Partitioning of the DEGs was performed by using the k-means method. Expression profiles of DEGs were displayed as false-colour heatmaps using the ‘pheatmap’ package. Functional analyses were performed with AMEN (Chalmel and Primig, 2008) with an FDR-adjusted *P* ≤ 0.05.

## Results

### Trans tissue can form cytotypic and compartmentalized organoids

To investigate how trans tissue performs regarding organoid generation in comparison with testicular tissues at different stages of development, organoids were derived from prepub (n = 3), pub (n = 3), cis (n = 5), and trans (n = 11) tissues.

**Figure 1.**
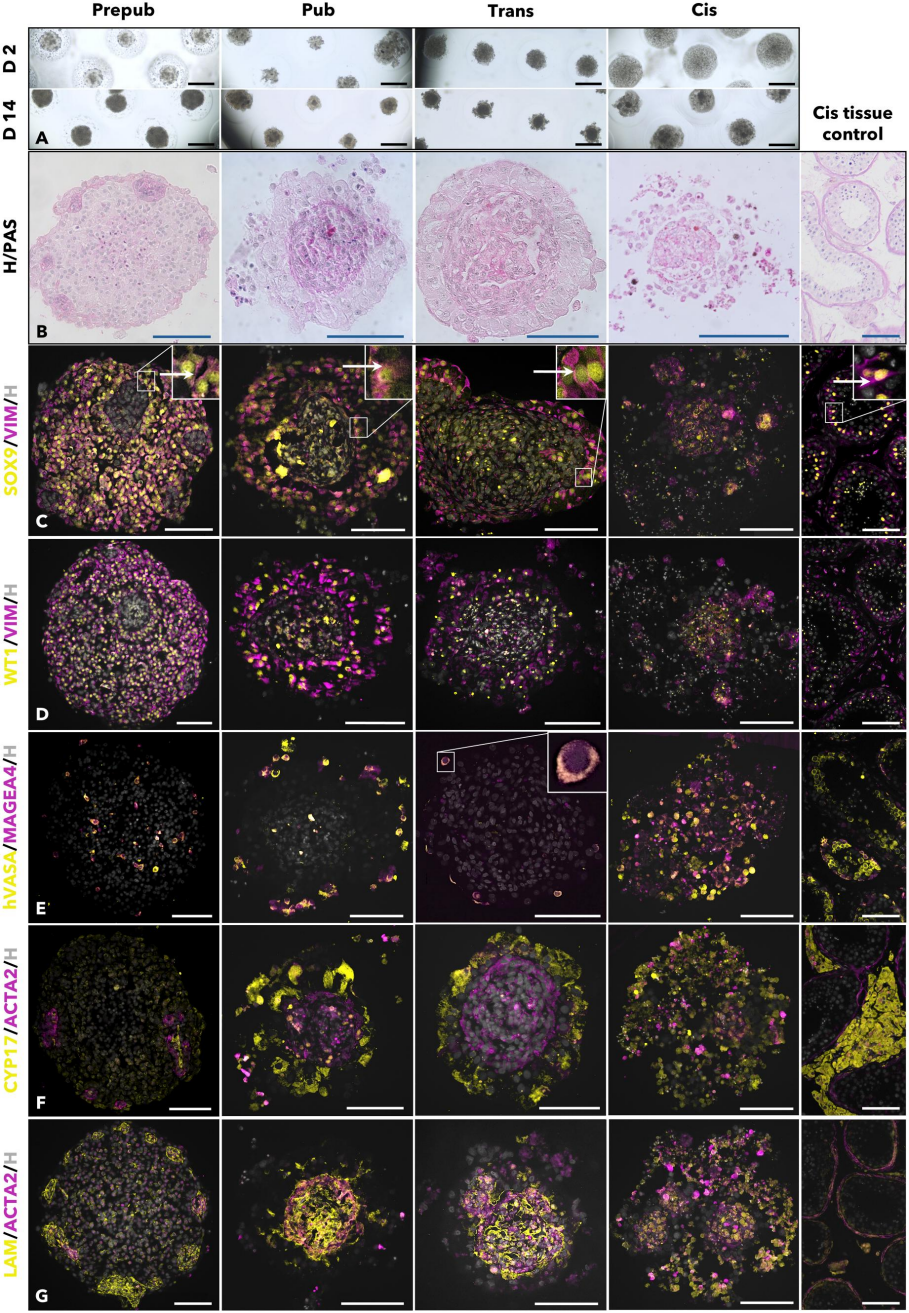
**Trans tissue can form cytotypic and compartmentalized organoids.** Histological comparison between prepubertal (prepub, n = 3), pubertal (pub, n = 3), transgender (trans, n = 11), and cisgender (cis, n = 5) tissue-derived organoids. (**A**) Representative pictures of organoids derived from the different tissues at Days 2 and 14 in culture in 3D Petri Dishes^®^. Scale bar: 500 μm. (**B**) H/PAS-stained organoid and control tissue sections to show general morphology at Day 14. Scale bar: 100 μm. (**C–G**) Organoid and control tissue sections show presence of important testicular markers at Day 14: (C) SOX9/VIM (arrow points to elongating VIM pattern), (D) WT1/VIM, (E) hVASA/MAGEA4, (F) CYP17/ACTA2, (G) LAM/ACTA2. SOX9, SRY-box transcription Factor 9; VIM, vimentin; WT1, Wilm’s tumour gene 1; hVASA, human VASA; MAGEA4, melanoma-associated antigen 4; CYP17, cytochrome P450 17A1; ACTA2, actin alpha 2; LAM, laminin. Scale bar: 100 μm. H, Hoechst nuclear staining.

Cells from prepub, pub, and trans tissues aggregate and compact over 14 days in culture, whereas cells from cis tissue aggregate less and show very little compaction. It was observed that cis tissue is the only cell source that does not allow organoid organisation. In contrast, trans and pub tissues form a bicompartmental architecture composed of a dense core filled with extracellular matrix (ECM) filaments and cells and an outer compartment populated by cells in a loser arrangement. Cells from prepub tissue, however, organize into a more homogeneous configuration with scattered clusters of cells intertwined by ECM filaments and apparent polarizing cells adhering to the outer lining of these clusters. Representative pictures of organoids on Days 2 and 14 in culture and sections of organoids on Day 14 can be found in Fig. 1A and B.

The organoids were immunofluorescently stained with markers for the main testicular cells and ECM (Fig. 1C–G). Negative control pictures can be found in Supplementary Fig. S2. Organoids derived from prepub tissue showed scattered structures containing ACTA2^+^ cells (myoid cells), ECM, and CYP17^+^ Leydig cells. However, CYP17^+^ cells could also be found dispersed throughout the organoid. SOX9^+^ and WT1^+^ Sertoli cells were found throughout the whole organoid except in the scattered structures. The Sertoli cells attached to the scattered structures showed an elongating vimentin pattern similar to that of polarized Sertoli cells attached to the basement membrane of seminiferous tubules. Germ cells (MAGEA4^+^ and hVASA^+^) were found between Sertoli cells throughout the organoid. The spaces between the scattered structures may be considered seminiferous areas where germ and Sertoli cells reside, with the latter polarizing when anchored. Regarding pub and trans organoids, Sertoli cells (SOX9^+^ and WT1^+^) could be found in the outer compartment. Those closest to the core of the organoids displayed polarization. SOX9^+^ and WT1^+^ cells could also be found in the core of the organoids, although SOX9 was often observed diffused in the cytoplasm instead of located in the nuclei. Germ cells (MAGEA4^+^ and hVASA^+^) were found in the outer compartment, usually on the outer edge. Leydig cells (CYP17^+^) could be found in the outer compartment mixed with Sertoli cells, in contrast with peritubular myoid cells (or other ACTA2^+^ cells), which were found in the core of the organoids, often displaying a more spindle-like shape. Laminin and other ECM proteins were also found in the core (Supplementary Fig. S3). In organoids derived from trans and pub tissues, the epithelium can be described as inside-out since it is not turned inwards, forming the core of the organoid, but rather oriented to the outside of the organoid. However, Leydig cells are still in the outer compartment, where they would be expected to closely mimic the testicular architecture. To rule out any potential effect of the cryopreservation over the trans-organoid architecture, organoids were also made from fresh tissue. However, no difference was seen between organoids derived from fresh or cryopreserved tissue (Supplementary Fig. S4). Although it was possible to derive spheroidal structures from all trans tissues, 4 out of 11 did not aggregate well or did not show the bicompartmental architecture (Supplementary Fig. S5). Long-term 63- (n = 3) and 100-day (n = 1) organoids showed increased compaction over time and displayed the same structure and cell types (Supplementary Fig. S6). Cis organoids were positive for all markers, but no specific location could be attributed to any cell type as they were not well organized, and laminin distribution was dispersed.

### AMH expression and duration of gender-affirming hormone therapy predict organoid formation

Various characteristics of the trans tissue donors were evaluated to identify potential predictors for the preferred organoid architecture. The preferred organoid architecture, further called ‘bicompartmental’ was observed in 7 of the 11 organoids (Tr3, Tr4, Tr7, Tr8, Tr9, Tr10, and Tr12). The remaining organoids exhibited other types of architecture, hereon called ‘irregular’ (Tr2, Tr5, Tr6, and Tr11).

**Figure 2.**
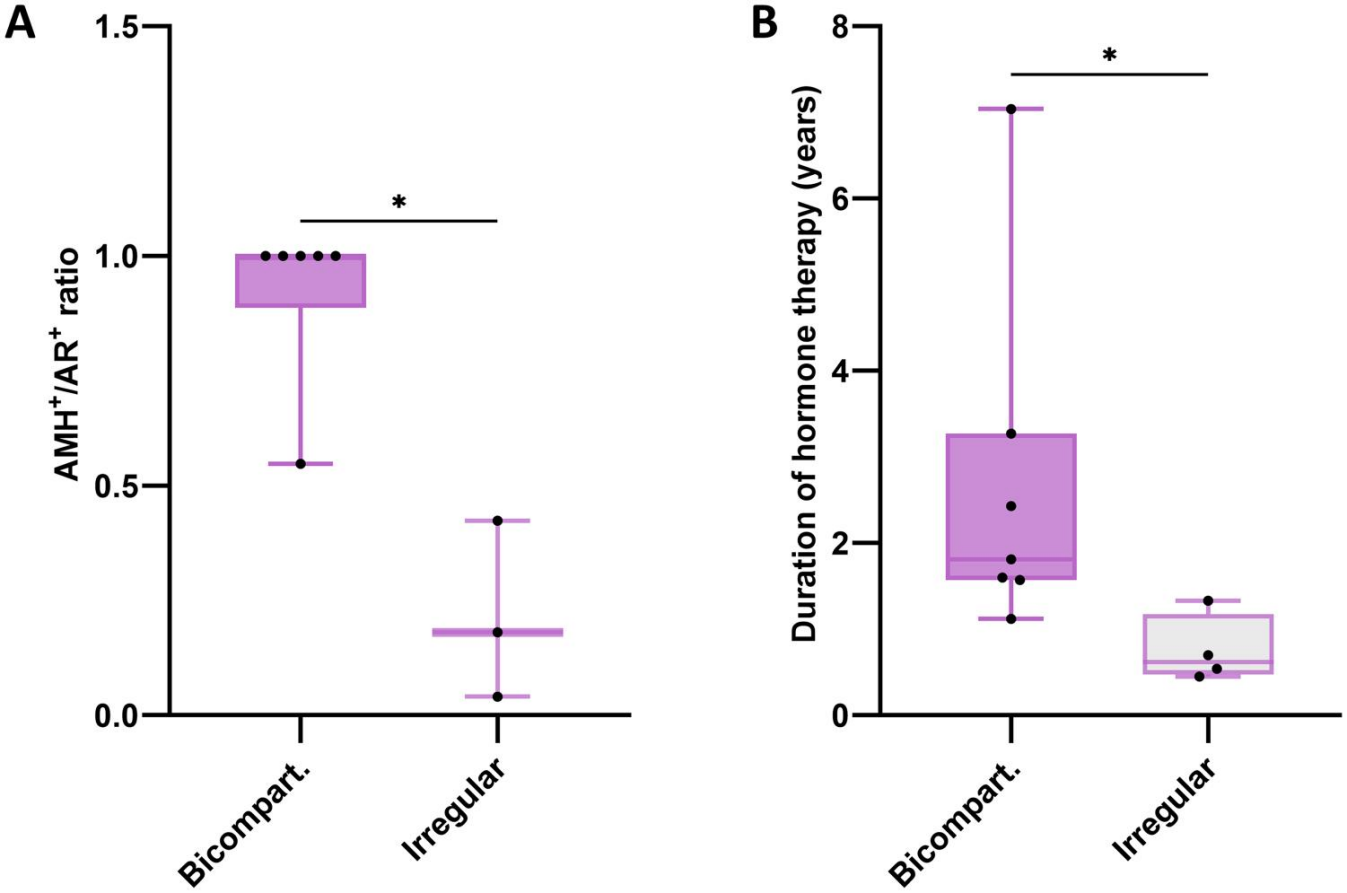
**Anti-Müllerian hormone expression and duration of gender-affirming hormone therapy predict organoid formation.** (**A**) Trans tissues with a high ratio of anti-Müllerian hormone (AMH)^+^ over androgen receptor (AR)^+^ seminiferous tubules form bicompartmental organoids. (**B**) Trans tissues exposed to hormone therapy for a longer than one year tend to form bicompartmental organoids. Bicompart, bicompartmental. For statistical evaluation, Mann–Whitney *U*-test was used. *P < 0.05.

Some tissues forming irregular organoids showed fewer seminiferous tubules compared to those forming bicompartmental organoids (Supplementary Fig. S5). Despite this, quantification of the area occupied by seminiferous tubules revealed no significant difference between the ‘bicompartmental’ and ‘irregular’ groups (Supplementary Fig. S7A). This indicates that the samples in the ‘irregular’ group were not consistent in histology, with either a varying number of tubules for the same area or larger ones. A lower ratio of SOX9^+^/WT1^+^ cells has been associated with limited organoid formation (Cui *et al.*, 2024); however, no statistical difference was detected in the present study (Supplementary Fig. S7B). Additionally, the ratio of AMH^+^/AR^+^ tubules was assessed based on previous findings showing that adult transgender seminiferous tubules could express both AMH and AR, only AMH, or only AR (Delgouffe *et al.*, 2025). The presence of AMH indicates that Sertoli cells have regressed in maturity, and that might influence organoid formation potential. Notably, a higher AMH^+^/AR^+^ ratio was found to significantly (*P *= 0.0119) correlate with the formation of bicompartmental organoids (Fig. 2A).

Analysis of donor age revealed no statistically significant difference between both groups (Supplementary Fig. S7C). Similarly, the groups were compared based on oestrogen and testosterone levels, but no significant difference could be found (Supplementary Fig. S7D and E). Since not all hormone levels were available for all donors, and the variability in the timing of measurements relative to the start of hormone therapy or orchidectomy limits the ability to establish correlations between hormone levels and organoid architecture, the duration of hormone therapy was examined as a more consistent variable. Tissues that gave rise to organoids with a bicompartmental architecture were associated with longer hormone therapy durations compared to those producing organoids with irregular architecture (*P *= 0.0121, Fig. 2B).

### Trans organoids produce testosterone levels comparable to prepub organoids

**Figure 3.**
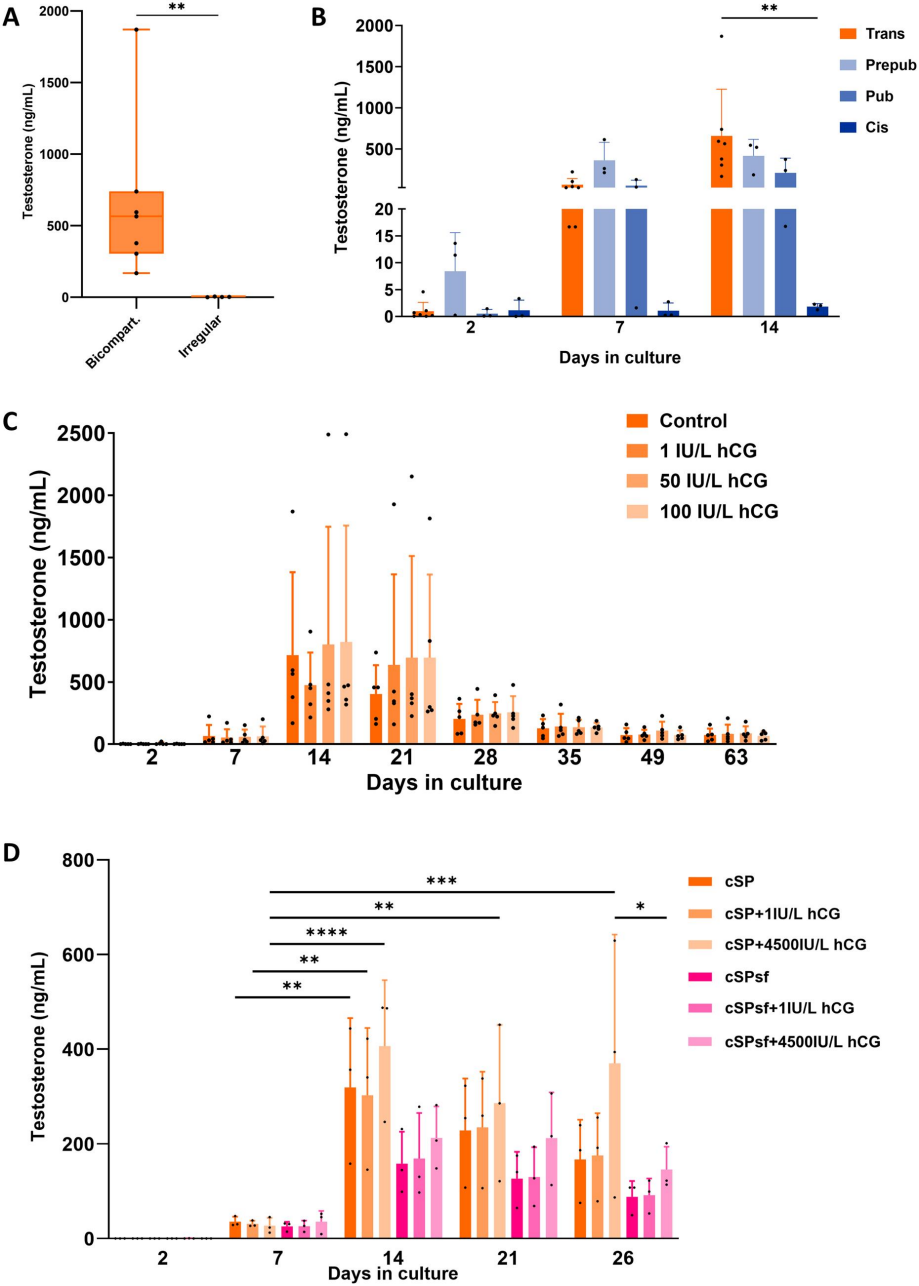
**Trans organoids produce testosterone for at least 2 months and are responsive to high levels of hCG.** (**A**) Bicompartmental (bicompart) organoids produce significantly higher levels of testosterone compared to irregular organoids. For statistical evaluation, a Mann–Whitney *U*-test was used. (**B**) Transgender (trans, n = 7), prepubertal (prepub, n = 3), pubertal (pub, n = 3), and cisgender (cis, n = 3) organoids were compared regarding testosterone production over a culture period of 14 days. For statistical evaluation, an ANOVA followed by a Tukey’s multiple comparisons test was used. (**C**) Testosterone production by trans organoids (n = 5) was evaluated over 63 days and exposed to 1, 50, and 100 IU/l hCG. For statistical evaluation, an ANOVA followed by a Tukey’s multiple comparisons test was used. (**D**) Testosterone production by transgender tissue-derived organoids (n = 3) was evaluated over 26 days comparing complete StemPro (cSP) and cSP serum-free (sf) conditions without and with (1 or 4500 IU/l) hCG stimulation. For statistical evaluation, an ANOVA followed by a Tukey’s multiple comparisons test was used. *P < 0.05; **P < 0.01; ***P < 0.001; ****P < 0.0001.

At Day 14 of culture, a clear difference in testosterone production was observed between bicompartmental and irregular trans organoids (Fig. 3A). Irregular organoids exhibited low testosterone levels (2.5 ± 2.1 ng/ml), whereas bicompartmental organoids produced significantly higher levels (660.0 ± 567.1 ng/ml; *P *= 0.0061). Consequently, only bicompartmental organoids were included in subsequent testosterone analyses.

When compared with prepub, pub, and cis organoids, trans organoids demonstrated a production profile similar to prepub and pub organoids (Fig. 3B). At Day 14 in culture, trans organoids produced 660.0 ± 567.1 ng/ml of testosterone, while prepub organoids produced 418.0 ± 200.2 ng/ml, pub organoids produced 210.3 ± 180.3 ng/ml, and cis organoids produced 1.8 ± 0.5 ng/ml. Testosterone production in trans organoids at Day 14 was thus significantly higher than that of cis organoids (*P *= 0.0025).

### Testosterone production is maintained for at least 2 months in trans organoids

Following the observation of increasing testosterone production in trans organoids over the first 14 days, a culture experiment was set up (n = 5) to investigate how testosterone production would change over an extended period (Fig. 3C). Additionally, hCG was added at different concentrations to study whether it could support testosterone production in organoids over time. A physiologically relevant concentration of 1 IU/l was used [equivalent to 6–8 IU/l of LH, representing a normal serum concentration (Ezcurra and Humaidan, 2014)], as well as higher concentrations of 50 IU/l and 100 IU/l. Testosterone production peaked between Days 7 and 21 and stabilized at ≈73 ng/ml after Day 35. No significant differences in testosterone levels were observed between the control group and those treated with different concentrations of hCG.

### Trans organoids respond to high levels of hCG after 14 days in culture

Since KSR has been described as a testosterone-stimulating compound (Reda *et al.*, 2017), we compared testosterone production in trans organoids cultured in medium with KSR (cSP) and without KSR (cSPsf) for 26 days. Since KSR could be masking the effect of hCG, we re-exposed the organoids to 1 IU/l as well as to 4500 IU/l (Edmonds and Woodruff, 2020; Cham *et al.*, 2024) (Fig. 3D). While a statistically significant difference between cSP and cSPsf conditions was only found on Day 26 for trans organoids exposed to 4500 IU/l of hCG (*P *= 0.0384), testosterone levels were consistently lower under cSPsf conditions compared to their cSP counterparts. Despite the removal of KSR, 1 IU/l of hCG did not stimulate testosterone production above control levels. However, exposure to 4500 IU/l of hCG maintained testosterone levels at a significantly higher level than controls when compared to Day 7 testosterone levels.

### Cell proportions in trans organoids resemble those found in prepub and pub tissues

**Figure 4.**
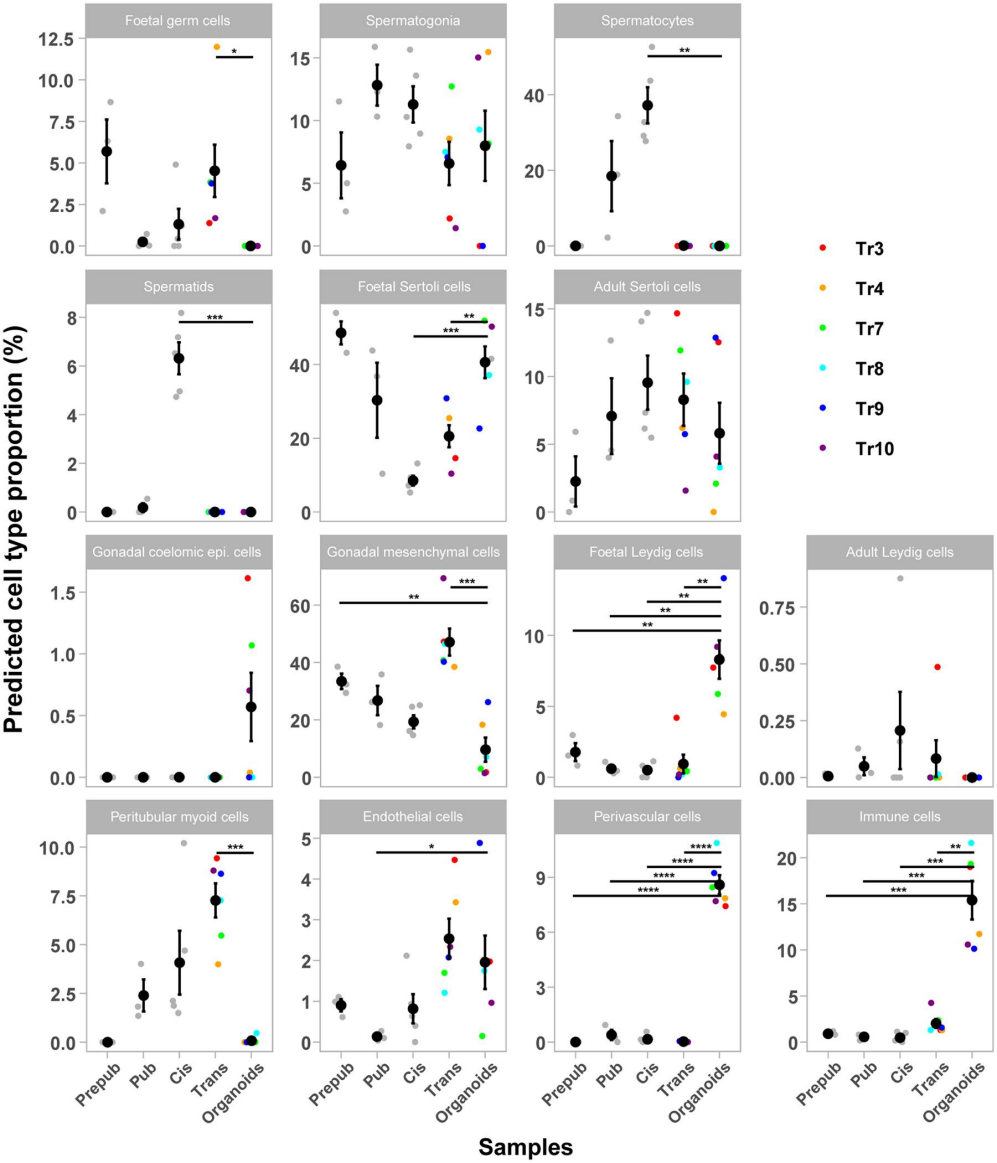
**Deconvolved bulk RNA-seq data showing cell populations and their predicted proportions.** Cell proportions are shown for in prepubertal (prepub, n = 3), pubertal (pub, n = 3), cisgender (cis, n = 5), and transgender (trans, n = 6) tissues, as well as bicompartmental organoids (n = 6). Cell types that were not represented in the samples or did not have any apparent biological relevance are not plotted. Each data point in a graph represents an individual sample. Trans tissue samples and the organoids they originate are colour-coded. Epi, epithelial. For statistical evaluations, the Student’s *t*-test implemented in R was used. *P < 0.05; **P < 0.01; ***P < 0.001; ****P < 0.0001.

To identify cell populations and proportions in trans bicompartmental organoids at Day 14 (n = 6)—hereafter referred to as ‘organoids’ in the results section—and their potential influence on architecture and testosterone production, bulk RNAseq data were deconvolved through scRNAseq data and compared to that of prepub (n = 3), pub (n = 3), cis (n = 5) tissues, as well as the trans tissues used to form the organoids (n = 6, Fig. 4). It is important to note that the cell population percentages obtained from deconvolution are only predicted proportions within a specific sample. Additionally, we use the terms ‘foetal’ or ‘adult’ to describe certain cell type subphenotypes, as the technique identifies transcripts in our bulk RNAseq data that are expressed in proper foetal or adult cells. This does not necessarily mean that the identified cells in the samples are *de facto* ‘foetal’ or ‘adult’, but rather that they have a phenotype that aligns with those in the scRNAseq data used.

In general, for the cell populations analysed, which include all the main testicular cells, organoids showed the greatest similarity to prepub and pub tissue samples (15 out of 19 evaluated cell types), followed by cis (13/19), and finally, trans (12/19) tissue samples.

Regarding seminiferous tubular cells, differences in cell proportions were found in both germ and Sertoli cells. Foetal germ cells were less prevalent in organoids compared to trans tissue (*P *= 0.0352). Spermatocytes and spermatids were absent in the organoids while present in cis tissue (*P *= 0.0015 and *P *= 0.0007, respectively). Foetal Sertoli cells were present at higher proportions in the organoids compared to cis and trans tissues (*P *= 0.0004 and *P *= 0.0041, respectively). Although not statistically significant, gonadal coelomic epithelial cells emerged in some organoids despite not being detected in any of the tissue samples. In mice, these are progenitor cells of Sertoli cells and interstitial cells, and might also be of peritubular myoid cells (Karl and Capel, 1998; Svingen and Koopman, 2013).

Significant differences were also observed in interstitial cell proportions. Gonadal mesenchymal cells had a significantly decreased cell proportion in organoids compared to trans tissue (*P *= 0.0001) and, to a lesser extent, prepub tissue (*P *= 0.002). Foetal Leydig cells were present in significantly greater proportion in the organoids than in any tissue sample (highest *P*-value—*P *= 0.0037). Although present in trans tissue, peritubular myoid cells disappeared in most organoids (*P *= 0.0004). Endothelial cell proportions increased significantly in the organoids compared to pub tissue (*P *= 0.0386). Additionally, perivascular and immune cell proportions increased significantly in the organoids compared to all tissue groups (highest *P*-values—*P *= 0.0001, *P *= 0.0011, respectively).

To summarize, organoids share the same tubular cell proportions with prepub and pub tissues while having an interstitial cell population that does not resemble any particular tissue. Table 1 summarizes the cell populations present in the organoids and their matched proportions across tissue samples.

**Table 1.** Summary of cell proportions present in bicompartmental organoids matched to tissue samples.

Cell type	Organoids	Prepub	Pub	Cis	Trans
**Spermatogonia (%)**	8.0 ± 6.3	6.4 ± 3.7	12.8 ± 2.3	11.3 ± 2.9	6.6 ± 3.8
**Foetal Sertoli cells (%)**	40.6 ± 9.6	48.6 ± 4.4	30.3 ± 14.4	**8.5 ± 2.7**	**20.5 ± 6.7**
**Adult Sertoli cells (%)**	5.8 ± 5.0	2.3 ± 2.6	7.1 ± 4.0	9.6 ± 4.0	8.3 ± 4.3
**Gonadal coelomic epithelial cells (%)**	0.6 ± 0.6	0.0 ± 0.0	0.0 ± 0.0	0.0 ± 0.0	0.0 ± 0.0
**Gonadal mesenchymal cells (%)**	9.6 ± 9.4	**33.4 ± 3.8**	26.7 ± 7.2	19.3 ± 4.5	**47.1 ± 10.5**
**Foetal Leydig cells (%)**	8.3 ± 3.0	**1.8 ± 0.9**	**0.6 ± 0.3**	**0.5 ± 0.4**	**0.9 ± 1.5**
**Peritubular myoid cells (%)**	0.1 ± 0.2	0.0 ± 0.0	2.4 ± 1.2	4.1 ± 3.3	**7.3 ± 2.0**
**Endothelial cells (%)**	2.0 ± 1.5	0.9 ± 0.2	**0.1 ± 0.1**	0.8 ± 0.7	2.5 ± 1.1
**Perivascular cells (%)**	8.6 ± 1.2	**0.0 ± 0.0**	**0.4 ± 0.4**	**0.2 ± 0.2**	**0.0 ± 0.0**
**Immune cells (%)**	15.4 ± 4.7	**0.9 ± 0.2**	**0.6 ± 0.3**	**0.5 ± 0.5**	**2.0 ± 1.1**

Values represent mean predicted proportion ± SD. Bold values are statistically different from organoids’ means according to the statistical evaluations described in Fig. 4. Prepub, prepubertal; pub, pubertal; cis, cisgender; trans, transgender.

### Bicompartmental and irregular organoids show different cell proportions

**Figure 5.**
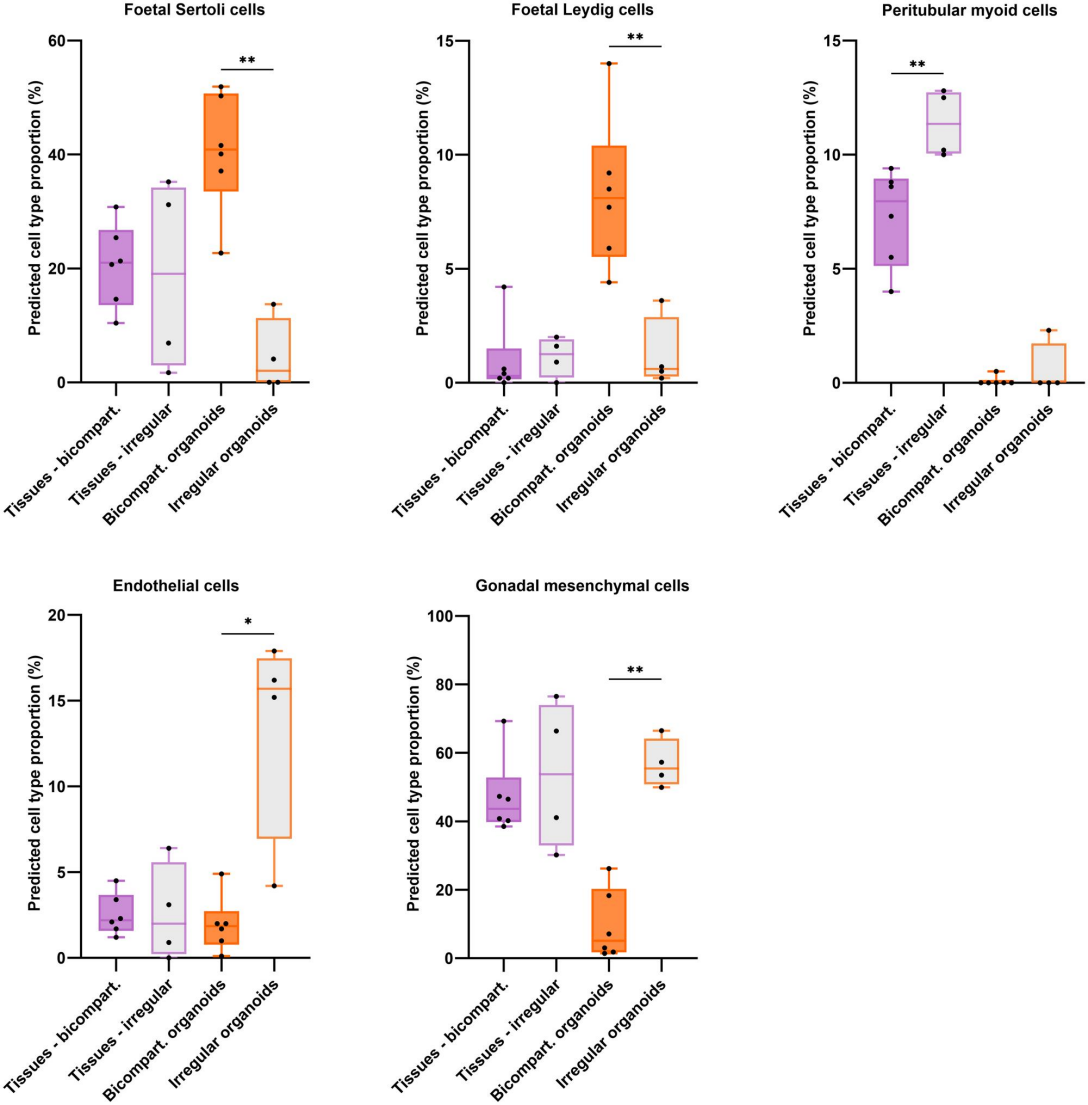
**Cell populations with significantly different cell proportions between transgender tissues generating ‘bicompartmental’ and ‘irregular’ organoids, or between ‘bicompartmental’ and ‘irregular’ transgender tissue-derived organoids.** Purple outlines: transgender tissues (n = 10). Orange outlines: transgender tissue-derived organoids (n = 10). For statistical evaluation, Mann–Whitney *U*-test was used. *P < 0.05; **P < 0.01.

Deconvolution was also performed on the irregular organoids to detect differences in cell proportions between the two organoid groups. Significant differences were found in the proportions of foetal Sertoli cells, foetal Leydig cells, endothelial cells, and gonadal mesenchymal cells (Fig. 5). Bicompartmental organoids exhibited significantly higher proportions of foetal Sertoli and Leydig cells compared to irregular organoids (*P *= 0.0095 for both comparisons). In contrast, endothelial cells and gonadal mesenchymal cells were present at lower proportions in bicompartmental organoids (*P *= 0.019 and *P *= 0.0095, respectively).

### Organoid architecture depends on the cell composition of the tissue from which it derived

To further investigate how variations in cell proportions might influence the ability of trans tissues to form bicompartmental organoids, deconvolution was also performed on the tissues that gave rise to the irregular organoids. Peritubular myoid cells were found at lower proportions in tissues generating bicompartmental organoids than those generating irregular ones (*P *= 0.0095, Fig. 5). For an overview of the cell proportion in each individual sample and how the inclusion of irregular organoids and their tissue of origin affects comparisons with other tissue groups, see Supplementary File S1 and Supplementary Fig. S8, respectively.

### Organoids at Day 14 are functionally related to prepub and trans tissues

**Figure 6.**
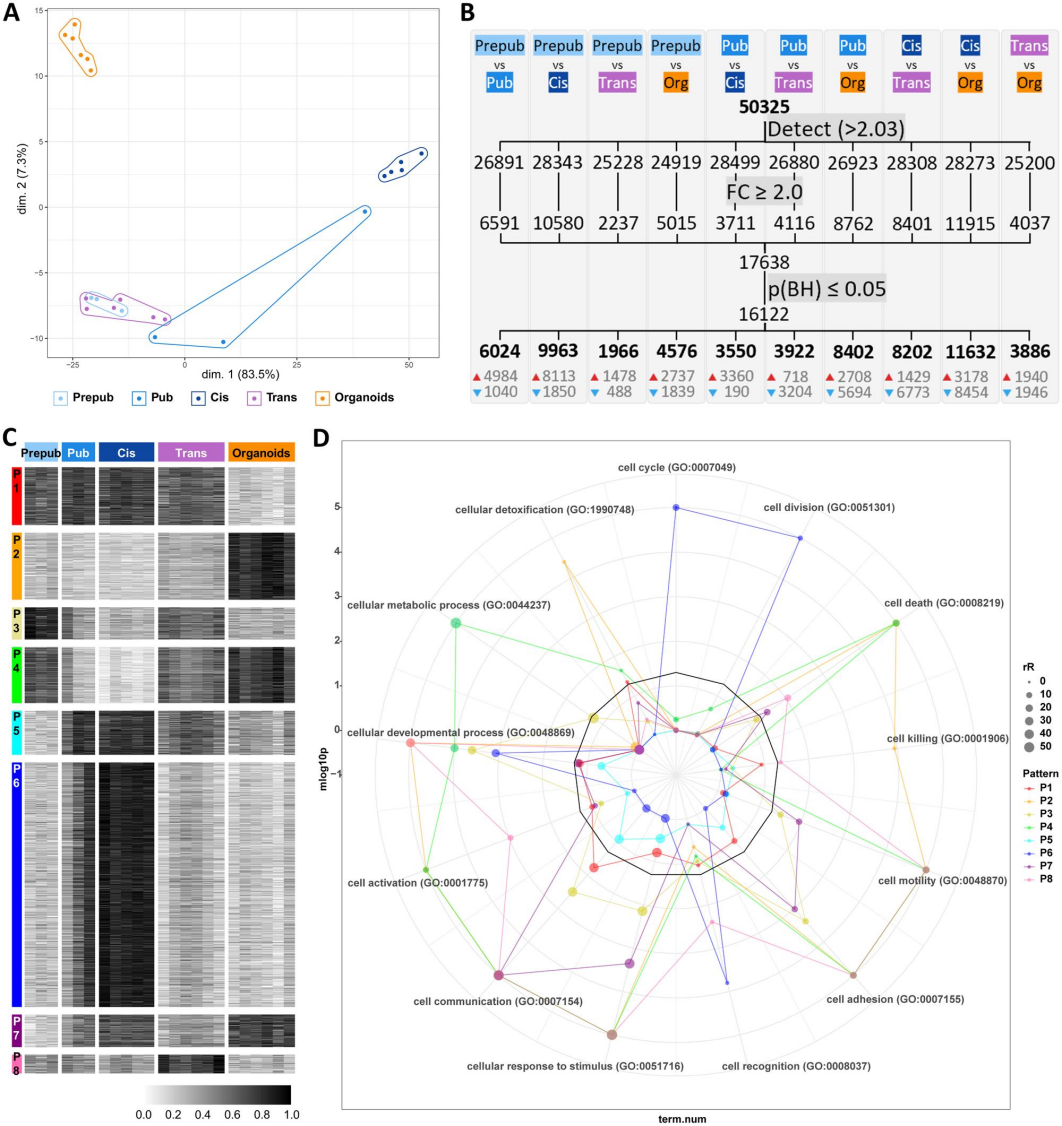
**Gene expression aligns transgender tissue-derived organoids with prepubertal and transgender tissues, while differentially expressed genes are shared with all tissue types.** (**A**) Two-dimensional PCA plot explaining the variation between tissue samples and organoids. (**B**) Filtration strategy for identification of differentially expressed genes (DEGs) between prepubertal (n = 3), pubertal (n = 3), cisgender (n = 5), transgender (n = 6) tissues, as well as bicompartmental organoids (n = 6). For each comparison, the number of genes above the background expression cutoff (2.032) is given (a total of 50 325 were analysed). The threshold for identifying DEGs was set to ≥2.0 fold-change (FC), and significance was determined using an adjusted *P*-value of ≤0.05. The numbers at the bottom of the columns show upregulated (red arrow) and downregulated (blue arrow) DEGs. BH, Benjamini and Hochberg. (**C**) DEGs clustered into eight expression patterns (P1–P8) and (**D**) plotted in a radar plot according to shared genes with each of 13 GO terms corresponding to a radius of the graph (see Supplementary Fig. S10 for the relationship between terms). When a pattern is over the cut-off line (−log_10_(0.05)≈1.3) for a GO term, the genes in that pattern are related to that GO term, and consequently, the samples with highest expression in that pattern are too. Sample types and expression patterns are consistently colour-coded throughout the figure. Prepub, prepubertal; pub, pubertal; cis, cisgender; trans, transgender; org, organoids.

Prepub (n = 3), pub (n = 3), cis (n = 5), trans (n = 6) tissues, and bicompartmental organoids (n = 6) were evaluated through gene expression clustering and DEGs. After data normalization, the transcriptomes were projected onto a two-dimensional PCA-based space. The organoids formed a separate cluster. The first principal component, which accounted for approximately 83.5% of the variation (*x*-axis), separated the samples based on the progression of spermatogenesis and tissue maturity (Supplementary Table S1), aligning the organoids with prepub and trans tissues, along with the pub closest to the prepubertal state (pub 1, Fig. 6A). A correlation matrix comparing all samples pairwise is available in Supplementary Fig. S9.

When comparing the different sample groups, a total of 17 127 DEGs were identified (Fig. 6B). These DEGs were subsequently clustered into eight expression patterns (P1–P8) to assess how the organoids compared to the different tissue samples (Fig. 6C). Functional analysis revealed 2794 enriched GO terms significantly associated with the eight expression patterns (Supplementary File S2). Since cell-type proportions vary across sample groups, DEG analyses may be skewed. For most patterns, testicular-specific cellular processes (GO terms) were not enriched. As a result, the focus of this functional analysis was placed on general cellular processes rather than specialized testicular functions. The eight expression patterns were plotted on a radar plot, based on the overlap of their genes with those from 13 high-level GO terms, distributed along the plot’s radii (Fig. 6D). These GO terms represent distinct cellular processes, not child or descendant terms of each other, stemming straight from ‘cellular process’ (GO:0009987) except for ‘cellular detoxification’ (GO:1990748), which can also fall under ‘cellular response to stimulus’ (GO:0051716) (Supplementary Fig. S10).

P2 was significantly enriched in genes coding for proteins involved in ‘cellular detoxification’ (24 genes, *P *= 0.0009), with the organoid group expressing these genes most prominently, likely due to the *in vitro* culture conditions. P2 and P4 were significantly enriched in genes for ‘cell death’ (GO:0008219; 318 genes, *P *< 0.0001; and 242 genes, *P *< 0.0001, respectively), with organoids expressing genes from these patterns most markedly. This is possibly linked to ‘cell killing’ (GO:0001906), which contains genes enriched only in P2 (34 genes, *P *= 0.0024), where organoids exhibit the highest expression. This term refers to one cell killing another, rather than a cell dying on its own, which may be associated with the elevated immune cell proportions in organoids, as seen in the deconvolution data—another potential consequence of *in vitro* culturing. Additionally, P2, P4, and P8 were significantly enriched in genes for ‘cell activation’ (GO:0001775; 190 genes, *P *< 0.0001; 126 genes, *P *< 0.0001; and 37 genes, *P *= 0.0374, respectively). ‘Cell activation’ concerns the morphological or behavioural changes in cells resulting from exposure to an activating factor. Interestingly, these patterns were enriched for ‘cell activation’-related terms concerning the immune system, such as ‘leukocyte activation’ (GO:0045321), connected to genes enriched in P2 and P4 (170 genes, *P *< 0.0001; and 102 genes, *P *= 0.006, respectively).

P2, P4, and P8 were significantly enriched in genes for ‘cell motility’ (GO:0048870; 318 genes, *P *< 0.0001; 318 genes, *P *< 0.0001; and 242 genes, *P *< 0.0001, respectively) and ‘cell adhesion’ (GO:0007155; 256 genes, *P *< 0.0001; 204 genes, *P *< 0.0001; and 63 genes, *P *< 0.0001, respectively). Organoids, along with prepub, pub 1, and trans tissues, express the genes in these patterns the most. This suggests significant cell movement and tissue remodelling, which aligns with the organoid development process *in vitro*, as well as morphogenesis and development in immature testicular tissue and the remodelling of trans tissue (Delgouffe *et al.*, 2025). P2, P4, and P8 were also significantly enriched in genes involved in ‘cellular response to stimulus’ (847 genes, *P *< 0.0001; 676 genes, *P *< 0.0001; and 208 genes, *P *< 0.0001, respectively) and ‘cell communication’ (GO:0007154; 752 genes, *P *< 0.0001; 621 genes, *P *< 0.0001; and 201 genes, *P *< 0.0001, respectively). P2 was characteristic of the organoids, while P8 was more specific to trans tissues. However, in P4, the organoids shared highly expressed genes with prepub, pub 1, and trans tissues, highlighting functional similarities. P2, P4, and P8 were still significantly enriched in genes involved in ‘cellular developmental process’ (GO:0048869; 511 genes, *P *< 0.0001; 384 genes, *P *= 0.0038; and 135 genes, *P *< 0.0001, respectively), suggesting that organoids, along with prepub, pub 1, and trans tissues are active in differentiation/dedifferentiation processes. In fact, P4, which connects all these sample groups, is enriched for genes involved in ‘cell differentiation’ (GO:0030154; 384 genes, *P *= 0.0024), ‘regulation of cell differentiation’ (GO:0045595; 171 genes, *P *< 0.0001), ‘mesenchymal cell differentiation’ (GO:0048762; 40 genes, *P *= 0.0005), and ‘epithelial cell differentiation’ (GO:0030855; 79 genes, *P *= 0.0152). Lastly, P4 was also significantly enriched in genes for ‘cellular metabolic process’ (GO:0044237; 815 genes, *P *= 0.0005), which refers to the biochemical pathways occurring within the cells.

P6 was significantly enriched in genes for ‘cell cycle’ (GO:0007049; 343 genes, *P *< 0.0001) and ‘cell division’ (GO:0051301; 130 genes, *P *< 0.0001), with cis tissue showing the highest expression, likely due to ongoing spermatogenesis.

Overall, while organoids show unique expression patterns, they also share expressed genes with prepub, pub 1, and trans tissues, indicating a functional similarity between them. Moreover, *in vitro* culture artefacts are apparent when comparing organoids to tissue samples.

## Discussion

Finding a reliable source of human testicular tissue for *in vitro* testis modelling is challenging, especially for applications requiring more than only a few thousand cells or a few cubic millimetres. However, the demand for human-based tissue models is growing as more relevant data for human reproductive biology or clinical research is needed, and regulatory agencies worldwide face increasing pressure to move away from animal testing (European Parliament and Council, 2021; US Food and Drug Administration, 2021; European Commission, 2023; Hansell *et al.*, 2024). While human stem cells have been explored for generating different types of testicular cells (Eguizabal *et al.*, 2011; Easley *et al.*, 2012; Gutiérrez *et al.*, 2018; Ishida *et al.*, 2021; Zhang *et al.*, 2021) or mimicking gonad development (Knarston *et al.*, 2020; Pryzhkova *et al.*, 2022), protocols remain underdeveloped, with cells often retaining an immature, foetal phenotype. This limits their applicability in modelling mature organ functions but makes them more suitable for organogenesis studies. In contrast, cells from primary tissue better preserve the epigenetic state of the original organ, maintaining specialized functions and cellular identity (Kim *et al.*, 2010; Ivanov *et al.*, 2016; Scesa *et al.*, 2021). Moreover, structures derived from stem cells, even if with diverse cell types, fail to fully replicate the cellular diversity of native tissue. Testis modelling protocols often focus only on the generation of haploid cells from germline-like stem cells alone, occasionally in co-culture with Sertoli-like cells, or the generation of Leydig-like cells for steroidogenesis study purposes, typically in 2D culture or in 3D configuration using Matrigel^®^ or other materials that are not suitable for clinical applications or substance testing. Therefore, in this study, we aimed to generate testicular organoids from human tissue following an easy-to-use and high-throughput-friendly protocol previously developed for mouse tissue (Richer *et al.*, 2024). The focus was placed on generating testicular organoids from trans tissue, which offers a unique opportunity due to its availability as frequent medical waste and regressed maturity (Delgouffe *et al.*, 2025). Although adult transgender cells have been used in 2D culture for toxicity testing (Mincheva *et al.*, 2020), such methods lack the scalability and standardization needed for broader applications. Here, we highlight the potential of trans tissue for developing robust and scalable testicular organoids.

This study confirms that adult/mature human testicular cells have very limited capability in forming organoids, as described with mouse and human (Baert *et al.*, 2017; Edmonds and Woodruff, 2020). In contrast, prepubertal testicular cells are more effective in establishing organoids, as shown with mouse, rat, pig, monkey, and human (Alves-Lopes *et al.*, 2017; Sakib *et al.*, 2019, 2022; Cham *et al.*, 2021; Richer *et al.*, 2021, 2024). Following a similar culture setup to ours, except for the use of Matrigel^®^, Sakib *et al.* (2019) formed compartmentalized organoids from human prepub tissue with GATA4^+^ Sertoli cells in an outer compartment and a core filled with ACTA2^+^ cells. However, this organization is not ideal if the aim is to have a model where the seminiferous epithelium, especially differentiating germ cells, is protected from outside insults by a ‘blood–testis barrier’. Such structures with an interstitial-like outer compartment and a seminiferous-like core were attained by our group using prepubertal mouse tissue (Richer *et al.*, 2021, 2024). As the cell seeding density used to achieve the desired architecture was higher than the one used by Sakib *et al.* (2019), we hypothesized that if more human testicular cells were seeded per organoid, similar structures would be generated as in our mouse studies. However, that was not fully the case. The basement membrane was not formed uninterruptedly around an epithelial structure in the human prepub organoids. Instead, several scattered structures that contained Leydig cells and resembled a stroma lined by a basement membrane were observed with an epithelial-like organization around them. However, these two interstitial and epithelial compartments already represent a good model if prepub tissue is available. Since the focus of this study was not on optimizing culture conditions for prepub organoids, we do not know if the structure of the prepub organoids could be further improved to more closely resemble seminiferous structures.

Most trans organoids formed bicompartmental structures, suggesting that the cells in the core aggregate faster than the epithelial ones on the outside. Alternatively, these cells might migrate to the middle after aggregation, with epithelial cells organizing on top. Regardless, Leydig cells are placed in contact with the medium, similarly to their location in the interstitium—the vascular compartment of the testis. Pub organoids followed the same architecture, confirming our previous findings that trans tissue has dedifferentiated to a partially immature state due to the hormone therapy (Delgouffe *et al.*, 2025). In these organoids, SOX9 expression was often observed in the cytoplasm. Interestingly, when a human testis-derived cell line was treated with oestrogen, an increase in cytoplasmic SOX9 expression was recorded (Stewart *et al.*, 2020). This mechanism might be at play in the organoids, since the trans tissue has been exposed to elevated levels of oestrogen, and the culture medium composition also includes oestrogen. Also noteworthy, organoids derived from fresh tissue exhibited the same architecture as those derived after cryopreservation, showing that the cryopreservation protocol maintains cell functionality effectively, as noted previously (Baert *et al.*, 2015). This is particularly relevant for the potential use of cryopreserved prepub tissue in restoring fertility after childhood cancer (Goossens *et al.*, 2020).

The bicompartmental architecture, however, was not always achieved in organoids from all trans tissues. Although there was always some degree of cell compaction compared to cis organoids, organoids from 4 of the 11 tissues had different architectures. Thus, we divided the trans tissues into two groups based on the organoid architecture they generated—‘bicompartmental’ or ‘irregular’. As Sertoli cells are known to be key drivers of seminiferous tubule formation during testis development (O’Donnell *et al.*, 2022), we looked at some Sertoli cell-specific markers and their ratios. A lower ratio of SOX9^+^/WT1^+^ Sertoli cells has been associated with a worse outcome in human prepub organoid formation and size (Cui *et al.*, 2024). However, we did not find any differences between the tissues. The changes found in SOX9^+^/WT1^+^ in the literature were associated with exposure to alkylating agents, which explains why we could not find any relevant differences. Based on the finding that most trans tissues express AMH and AR simultaneously in the seminiferous epithelium (Delgouffe *et al.*, 2025), we looked at the ratio of AMH^+^/AR^+^ tubules. Interestingly, the ‘bicompartmental’ group had more tubules expressing AMH, proving that immature Sertoli cells are crucial in forming organoids. Relating to oestradiol and testosterone serum levels, we found no differences between both groups. Although the gender-affirming hormone therapy aims to keep oestradiol levels between 100 and 200 ng/l and testosterone levels below 50 ng/dl, unfortunately, this analysis cannot be fully trusted because the time between when the hormone levels were assessed and the date of the orchidectomy varies widely. Some of the trans women stopped the hormone therapy 2 weeks before undergoing orchidectomy. During this period, when testosterone is not being actively blocked, serum levels may increase significantly (de Nie *et al.*, 2023). The positive outlier observed in the ‘bicompartmental’ group in the analysis correlating testosterone levels with the organoid architecture is the only one for which testosterone levels were measured within the therapy withdrawal before surgery. Nonetheless, the tissues in the ‘irregular’ group had a significantly lower therapy duration than those in the ‘bicompartmental’ group. It has been described that longer hormone therapies correspond to higher intratesticular AMH levels (Schneider *et al.*, 2021), which connects the observed significance in AMH^+^/AR^+^ ratio between both groups to the significance attributed to hormone therapy duration. Interestingly, the tissue that had the longest exposure to hormone therapy from the ‘irregular’ group (Tr11) gave rise to the organoid with the closest architecture to the bicompartmental ones. However, a difference between both adult transgender groups could not be observed regarding foetal or adult Sertoli cell proportions. It is important to note that although hormone therapy duration and Sertoli cell immaturity correlate with successful organoid formation, further studies are needed to establish causation.

The cessation of hormone therapy before orchidectomy could also influence organoid architecture. This could not be properly assessed due to the small sample size. However, we do not expect it to influence organoid architecture, as both bicompartmental and irregular organoids were obtained from donors who stopped hormone therapy 2 weeks prior to orchidectomy or those who did not.

Irregular organoids produced very low levels of testosterone, unlike bicompartmental ones. Since one potential application for the trans organoids is testing endocrine activity, irregular organoids were excluded from all other testosterone-related experiments. To help standardize the outcome of organoid generation from trans tissue and its testosterone competency, based on our results, only tissues from trans women treated following the World Professional Association for Transgender Health guidelines (Coleman *et al.*, 2022) for at least 1 year should be considered.

Compared to the other organoid types, trans organoids produced the highest levels of testosterone at Day 14. This contrasts with the cis organoids, which produced very little testosterone. On the other hand, they are in line with the prepub and pub organoids, which also showed an increase in testosterone production from Days 2 to 14. Although this seems counterintuitive considering the testosterone production of the testicular tissues of origin (no production during prepuberty, with increasing production from puberty until adulthood, where it remains stable until decreasing later in life), others have shown the same (Baert *et al.*, 2017; de Michele *et al.*, 2018; Medrano *et al.*, 2018; Richer *et al.*, 2021; Aden *et al.*, 2023; Cui *et al.*, 2024), suggesting an accelerated maturation or activation of non-androgenic Leydig cells *in situ* when placed *in vitro*. Again, similar to human immature tissue cultures, testosterone production in trans organoids reaches the highest levels between Days 7 and 21 in culture (de Michele *et al.*, 2018; Medrano *et al.*, 2018; Aden *et al.*, 2023). After this peak, levels gradually decrease and stabilize, aligning with two reports on long-term human immature testicular tissue cultures (de Michele *et al.*, 2018; Medrano *et al.*, 2018).

In a study culturing human immature testicular tissue with 5 IU/l of LH [equivalent to approximately 1 IU/l of hCG (Ezcurra and Humaidan, 2014)], LH had no significant effect on testosterone production (Medrano *et al.*, 2018). Interestingly, temperature and the addition of FBS or KSR were the determinants for an earlier or higher peak in testosterone production. In fact, KSR has been directly linked to increased testosterone production in mouse testicular tissue cultures (Reda *et al.*, 2017). Therefore, we hypothesized that the addition of KSR to the medium might mask potential hCG stimulation in the culture. As KSR’s main component is AlbuMAX^®^ I, which is rich in cholesterol and other lipids (Price *et al.*, 1998; Sanjo *et al.*, 2018), and steroidogenesis is fuelled by cholesterol, this may explain the advantage of the cSP condition over cSPsf in testosterone production at Day 26 with 4500 IU/l of hCG. The lack of statistically significant differences between other conditions could be due to the small number of replicates and high variability in testosterone production between trans organoids from different tissues. When focusing on the effect of hCG stimulation, the 1 IU/l dose had no effect on testosterone production in the cSPsf condition. Literature suggests that a distinct change in testosterone production is only seen as a result of hCG stimulation at an apparent physiologically irrelevant concentration of 4500 IU/l (Edmonds and Woodruff, 2020; Cham *et al.*, 2024). Despite no statistically significant difference between non-stimulated and 4500 IU/l-stimulated conditions within each time point, we can still observe a tendency for higher testosterone levels in the latter. However, looking at statistically significant differences over time, the cSP condition stimulated with 4500 IU/l of hCG is the only one showing consistently higher testosterone levels than on Day 7. The high variability in testosterone production levels between organoids derived from different patients hinders stronger statistical power. Nevertheless, the testosterone production profile over time is similar across cultures of different patients.

High proportions of foetal Leydig cells in trans organoids at Day 14 could explain the high testosterone levels at the same time point. In mice, foetal Leydig cells are LH independent (Zirkin and Papadopoulos, 2018; Bhattacharya and Dey, 2023), but this has not been established in humans. They respond to hCG *in utero* and to LH straight after birth, but whether this is the only way they are activated is not clear. However, the deconvolution data confirm that foetal Leydig cells remain in the testis throughout life (Shima, 2019) and can be activated in culture. The progenitor(s) of both foetal and adult Leydig cells are described as stem cells with mesenchymal-like appearance (Bhattacharya and Dey, 2023). Interestingly, gonadal mesenchymal cells are present in high proportions in trans tissue but disappear in Day-14 organoids. The decrease in testosterone over time in organoid cultures while these become sensitive to hCG suggests that Leydig cells may shift from a gonadotrophin-independent to a more dependent phenotype as the culture progresses.

Gonadal coelomic epithelial cells also appear in the organoids as progenitor cells, indicating possible epithelial-to-mesenchymal and mesenchymal-to-epithelial transitions supporting the forming structures. These are essential processes in gonadal development (Martinovic *et al.*, 2017).

It is interesting to observe that foetal germ cells decreased significantly in proportion from trans tissue to the organoids at Day 14. This decrease may indicate they die in culture or start differentiating into pro-/spermatogonia, although no significant increase was observed in spermatogonia from trans tissue to organoids. Differentiating primordial germ cells into spermatogonia is challenging in monocultures (Martin-Inaraja *et al.*, 2021); however, 3D cultures containing other testicular cells may facilitate this process. Although no effort was made at this point to support or induce spermatogenesis in the organoids, the maintenance of spermatogonia for 14 days indicates potential for differentiation with subsequent medium composition changes that support germ cell differentiation (Richer *et al.*, 2024). Moreover, a physiological 74-day spermatogenesis cycle can be planned since the organoids can be kept for at least 100 days. While spermatogonia were not clearly detected in the 63-day cultures, one was observed in the stained sections of the 100-day culture, similar to findings in long-term human testicular tissue cultures (de Michele *et al.*, 2018).

The steep drop in peritubular myoid cell proportion from trans tissue to organoids could be due to cell death in culture or phenotype transformation, changing cell transcripts and missing the identification through deconvolution. It is known that Sertoli cells are required to maintain the differentiated phenotype of peritubular myoid cells (Rebourcet *et al.*, 2014). However, in the organoids, Sertoli cells may not maintain all aspects they would in a well-established native architecture. Also, in mice, peritubular myoid cells have been proposed as sharing progenitors with Leydig cells (Ademi *et al.*, 2022) and could be dedifferentiating into progenitor cells to originate other cell types like Leydig cells. Interestingly, perivascular cells have followed the opposite trend, increasing in proportion from trans tissue to organoids. In mice, these cells can differentiate into Leydig cells but also smooth muscle cells (Kumar and DeFalco, 2018; Heinrich and DeFalco, 2020). Therefore, peritubular myoid cells may be dedifferentiating into a progenitor cell type rather than simply disappearing.

Immune cells are present in higher proportions in trans tissue and increase even more significantly in organoids, likely due to *in vitro* culturing inducing inflammation and cell death. This phenomenon, observed in testicular organ cultures (Abe *et al.*, 2020; Suzuki *et al.*, 2021) and supported by the gene expression functional analysis, may need to be addressed in future cultures to improve organoid architecture and promote an adequate environment for spermatogenesis (Abe *et al.*, 2020; Richer *et al.*, 2021, 2024; Suzuki *et al.*, 2021).

The analysis regarding cell proportions between ‘bicompartmental’ and ‘irregular’ organoid architectures showed higher proportions of foetal Sertoli and foetal Leydig cells in the bicompartmental organoids. This may explain the architectural differences—when thinking of foetal Sertoli cells—and increased steroidogenesis activity—when looking at foetal Leydig cells—between both groups. Conversely, gonadal mesenchymal cells decreased in bicompartmental organoids compared to irregular ones and the tissues of origin, emphasising that these cells may be differentiating into other cell types like foetal Leydig cells.

Peritubular myoid cells were the only cell type significantly different between trans tissue groups and thus the only cell-type predictor of a ‘bicompartmental’ or ‘irregular’ organoid architecture. The higher proportion of peritubular myoid cells in the ‘irregular’ tissue group may result from a decrease in other cell populations that, although not significant, when pulled together, increase the proportion of peritubular myoid cells. Moreover, since ACTA2 expression is stimulated by androgens (Schlatt *et al.*, 1993), shorter exposure to anti-androgen agents may maintain the peritubular myoid cell phenotype, as was previously observed (Delgouffe *et al.*, 2025), while longer exposure may lead to phenotype loss or dedifferentiation.

A limitation of the deconvolution data is that only cell proportions can be compared, not absolute numbers. Moreover, the accuracy of the technique depends on the quality of the reference datasets used. However, some findings corroborate aspects of the DEGs and functional analysis. Trans organoids share the most gene expression patterns and cellular processes with prepub and pub 1 tissue, as is evident in the shared cell type proportions. Additionally, the term ‘cell killing’, linked to genes enriched in organoids, relates to their high proportion of immune cells. Overall, while *in vitro* culturing artefacts differentiate organoids from tissues, gene expression analyses indicate that organoids at Day 14 may accurately mimic the testis in their varied cellular processes and stages, particularly the prepubertal testis. Since the expression patterns are generated based on the differences between the sample groups and were not primarily enriched in testicular-specific functions, this could corroborate this observation. However, this could also result from limitations of bulk RNA sequencing data. As cell-type proportions vary across and within sample groups, DEG analyses can be skewed. For more precise comparisons, single-cell sequencing should be considered.

Another limitation of this study is the limited number of prepubertal and pubertal tissue samples included due to the scarcity of these tissues for research. Although statistical significance could be obtained in comparisons with these two groups throughout the study, this limitation could affect the statistical power of comparisons.

To summarize, all major testicular cell types were detected through immunofluorescence in trans organoids, and Leydig cells were shown to be functional. When benchmarking Day-14 organoids against different testicular tissue maturity stages, the absence of *in vitro* spermatogenesis was confirmed. Spermatogonia and adult Sertoli cells were present in proportions similar to any tissue, while foetal Sertoli cells were similar to prepub and pub tissues only. Leydig cells were identified as foetal, and peritubular myoid cells were not detected. These last two cell types may relate to the disappearance of gonadal mesenchymal cells and the emergence of perivascular cells. The former could be an activated progenitor pool for foetal Leydig cells, explaining the testosterone surge around Day 14. The latter might result from peritubular myoid cells dedifferentiating once dissociated from their original location. Lastly, gene expression clustering revealed that Day-14 organoids closely match the profile of prepub and trans tissues. Interestingly, the functional analysis confirmed that organoids differed from the various tissues due to *in vitro*-induced changes, validating the technique.

Given their characteristics, trans organoids could be used for testing endocrine-disrupting chemicals. In fact, looking at their testosterone production profile, several crucial sensitive stages could be projected onto it. For instance, the first 14–21 days of culture can mimic the testosterone burst in the first 6–9 months of life during mini-puberty (Kurtoğlu and Baştuğ, 2014). If, however, one would be looking to test a chemical’s effect in adulthood, the more stable period after Day 35 would be better. In addition, the model has also the potential to test the effects of toxicants on Sertoli cells or spermatogonia. Eventually, if spermatogenesis is possible and the architecture is corrected, the effects of toxicants on spermatogenesis protected by a ‘blood–testis barrier’ can also be tested. Moreover, since the organoids are rich in different types of progenitor cells, differentiation studies could be performed. Clinically, as long as spermatogenesis is supported, they could host spermatogonia from infertile patients to generate gametes, even without a seminiferous tubular-like architecture. It is relevant to note that in this work, the cellular characterization of the organoids was focused on Day 14 of culture. However, as with testosterone production, the organoid cellular state might be rather dynamic than static, progressing from an immature to a more mature-like testicular state over time, opening many potential applications.

## Conclusion

This study demonstrates the successful generation of human testicular organoids from trans tissue, providing a novel and ethically sustainable human-based model for male reproductive health research, reproductive toxicology, and endocrine disruption studies. Trans organoids exhibit a bicompartmental architecture, present testicular cytotypic composition, and produce testosterone, closely resembling organoids derived from immature testicular tissues. Additionally, their cell composition is closest to prepubertal and pubertal tissue. Improved organoid formation is associated with longer hormone therapy, increased tubular expression of AMH, and a lower proportion of peritubular myoid cells in the tissue. These findings suggest that trans tissue, which is readily available and often discarded, can serve as a valuable resource for creating functional human testicular organoids.

## Supplementary Material

hoaf043_Supplementary_Data

## Data Availability

The bulk RNA-seq raw and preprocessed data supporting the findings of this study are stored under restricted access in the VUB Institutional Data Repository, under the identifier VUB/IVTD/1/000001, due to participant privacy concerns. Access to the data will be considered on a case-by-case basis and must be requested by contacting Prof. Dr Yoni Baert (yoni.baert@vub.be), who will review the request, including the intended purpose of the research and any potential commercial use. A data use agreement must be completed and signed following the VUB legal department’s guidelines before any data can be shared or released. Metadata for the dataset can be accessed through the VUB Research Portal at: https://researchportal.vub.be/en/datasets/testosterone-producing-and-cytotypic-human-testicular-organoids-w.
