# Supplementary material for: A new human in vitro model of cytotypic and testosterone-producing organoids derived from testicular tissue of transgender women
Source: Hum Reprod Open. 2025 Aug 20;2025(3):hoaf043. doi: 10.1093/hropen/hoaf043 (PMC12396852; doi:10.1093/hropen/hoaf043)
Supplement: hoaf043_Supplementary_Data [file hoaf043_supplementary_data.zip › Supplementary_Tables_S1-S3_&_Supplementary_Figures_S1-S10_EO.pdf]

Supplementary Table S1. Age of prepubertal, pubertal and cisgender tissue donors at the time of tissue collection and corresponding seminiferous epithelium description.

Sample name	Age at biopsy/ orchidectomy (years)	Histological notes
Prepub 1	1	Spermatogonia
Prepub 2	1	Spermatogonia
Prepub 3	4	Spermatogonia
Pub 1	13	Spermatocytes<br>Absent seminiferous lumen
Pub 2	15	Spermatocytes<br>Half-open seminiferous lumen
Pub 3	12	Elongated spermatids<br>Half-open seminiferous lumen
Cis 1	78	Normal spermatogenesis, signs of fibrosis
Cis 2	73	Normal spermatogenesis
Cis 3	72	Normal spermatogenesis
Cis 4	38	Normal spermatogenesis
Cis 5	84	Normal spermatogenesis

Prepub, prepubertal; pub, pubertal; cis, cisgender.

Supplementary Table S2. cSP medium composition.

Component	Concentration	Product number
StemPro™-34 serum-free medium (1x)	Basal medium	10639011, Gibco
StemPro™ supplement (40x)	2.5% (v/v)	10639011, Gibco
KnockOut™ Serum Replacement	10% (v/v)	10828010, Gibco
Antibiotic antimycotic solution (100X)	1% (v/v)	A5955, Sigma-Aldrich
MEM vitamin solution (100X)	1% (v/v)	11120052, Gibco
MEM Non-Essential Amino Acids Solution (100X)	1% (v/v)	11140050, Gibco
Insulin	25 µg/mL	I1882, Sigma-Aldrich
Transferrin	100 µg/mL	T1147, Sigma-Aldrich
Putrescine	60 µM	P7505, Sigma-Aldrich
Sodium selenite	30 nM	S5261, Sigma-Aldrich
D-(+)-glucose	6 mg/mL	G7021, Sigma-Aldrich
Pyruvic acid	30 µg/mL	P2256, Sigma-Aldrich
DL-lactic acid	1 µL/mL	L4263, Sigma-Aldrich
Bovine serum albumin	5 mg/mL	10735078001, Roche
L-glutamine	2 mM	25030024, Thermo Scientific
β-mercaptoethanol	50 µM	M6250, Sigma-Aldrich
Ascorbic acid	0.1 M	A7506, Sigma-Aldrich
Biotin	10 µg/mL	B4501, Sigma-Aldrich
β-oestradiol	30 ng/mL	E2758, Sigma-Aldrich
Progesterone	60 ng/mL	P8783, Sigma-Aldrich
Epithelial growth factor	20 ng/mL	PHGC311L, Gibco
Fibroblast growth factor 2	10 ng/mL	PMG0034, Gibco
Glial cell line-derived neurotrophic factor	10 ng/mL	PHC7045, Gibco

MEM, minimum essential media.

Supplementary Table S3. Antibody specifications.

Protein	Target	Ab type	Ab dilution	Reference
SRY-box transcription factor 9 (SOX9)	Sertoli cells	Rabbit pAb	1:200	AB5535, Sigma-Aldrich
Wilm's tumour gene 1 (WT1)	Sertoli cells	Rabbit mAb	1:200	AB89901, Abcam
Anti-Müllerian hormone (AMH)	Sertoli cells	Mouse mAb	1:200	MCA2246, BioRad
Androgen receptor (AR)	Sertoli cells	Rabbit mAb	1:200	AB133273, Abcam
Vimentin (VIM)	Sertoli cells	Mouse mAb	1:200	M0725, Dako
Human VASA (hVASA)	Germ cells	Goat pAb	1:100	AF2030, R&D Systems
Melanoma-associated antigen 4 (MAGEA4)	Spermatogonia	Mouse mAb	1:50	Provided by Dr. Giulio Spagnoli, University of Basel, Switzerland
Cytochrome P450 17A1 (CYP17)	Leydig cells	Rabbit pAb	1:400	14447-1-AP, Proteintech
Actin alpha 2 (ACTA2)	Peritubular myoid cells	Mouse mAb	1:400	A2547, Sigma-Aldrich
Fibronectin (FN)	Extracellular matrix	Rabbit pAb	1:800	A0245, Dako
Collagen type IV (COL4)	Extracellular matrix	Rabbit pAb	1:400	19674-1-AP, Proteintech
Laminin (LAM)	Extracellular matrix	Rabbit pAb	1:100	23498-1-AP, Proteintech
Anti-rabbit 488	Rabbit proteins	Donkey pAb	1:200	A-21203, Invitrogen
Anti-goat 488	Goat proteins	Donkey pAb	1:200	A-11055, Invitrogen
Anti-mouse 594	Mouse proteins	Donkey pAb	1:200	A-21206, Invitrogen
Anti-rabbit 488	Rabbit proteins	Goat pAb	1:200	A-11008, Invitrogen
Anti-rabbit HRP	Rabbit proteins	Goat pAb	1:200	PI-1000, Vector laboratories

Ab, antibody; mAb, monoclonal antibody; pAb, polyclonal antibody.

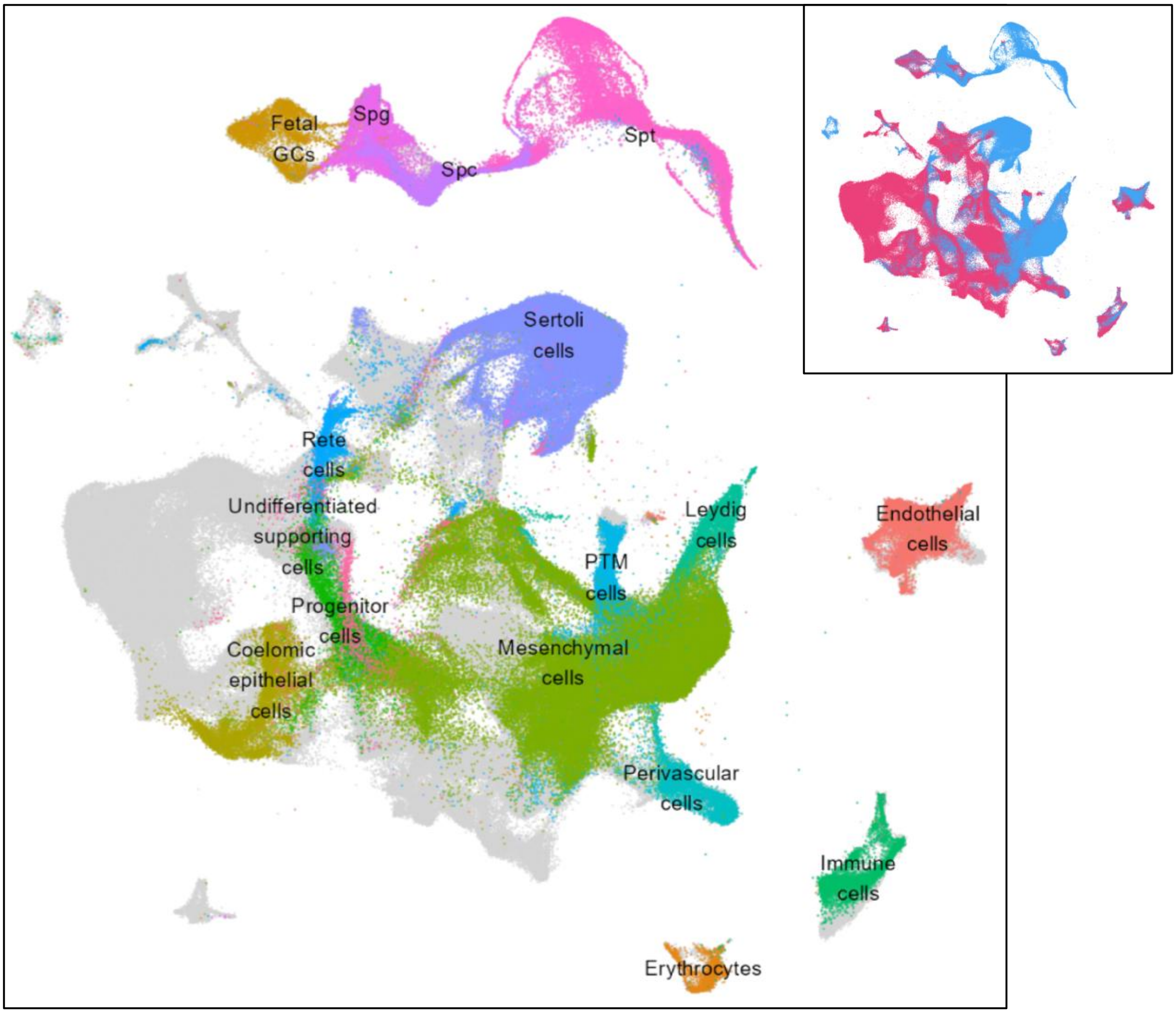

Supplementary Figure S1. Single-cell RNA-seq data-based maps of the developing human gonad. Accessible through the ReproGenomics Viewer database. The top-right panel displays a UMAP projection of gonadal cells, where pink areas represent ovarian cells and blue areas represent testicular cells. The large panel dissects the broad individual cell types used for deconvolution analysis in this study.

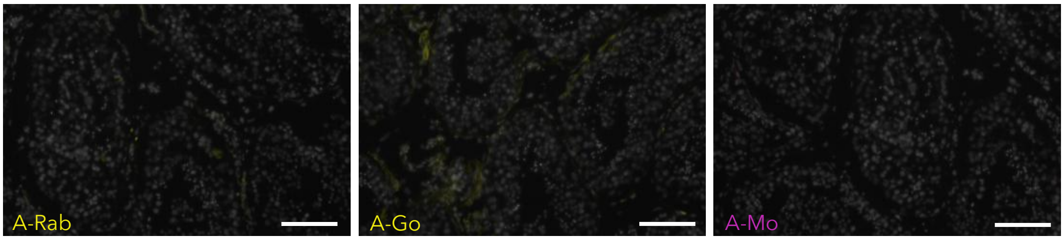

Supplementary Figure S2. Negative controls for immunofluorescence staining. Representative images showing the absence of non-specific signal in negative controls for secondary antibodies: anti-rabbit (A-Rb), anti-goat (A-Go), and anti-mouse (A-Mo). Scale bar: 100μm.

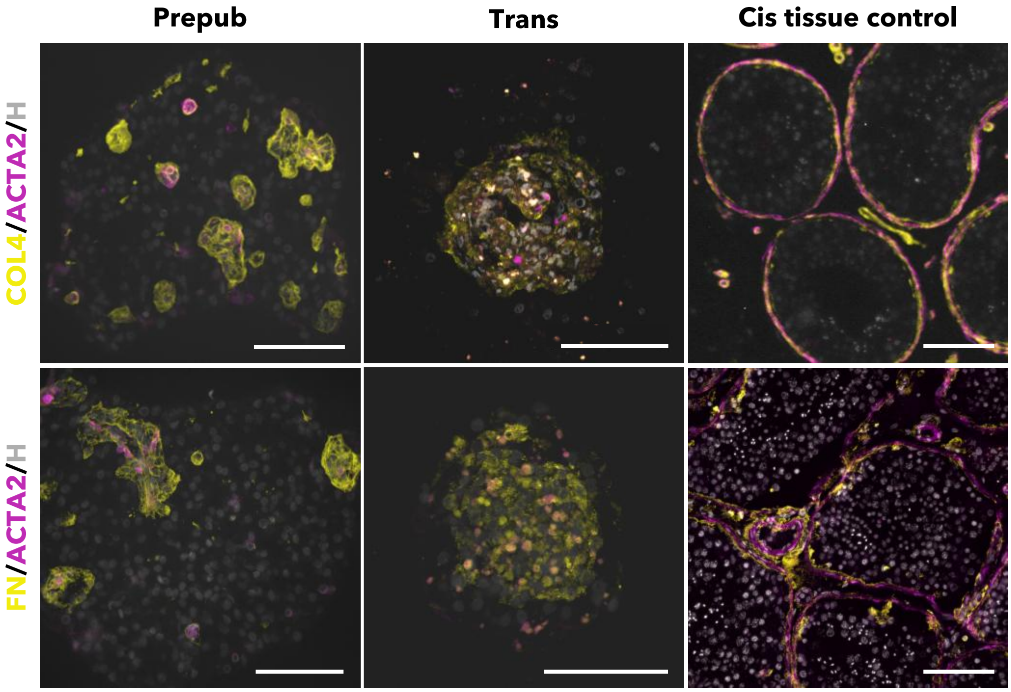

Supplementary Figure S3. ECM protein markers in prepubertal and transgender-tissue derived organoids at day 14. Immunofluorescent stainings of organoid cross sections at day 14. COL4, collagen type IV; FN, fibronectin; ACTA2, actin alpha 2. Prepub, prepubertal; trans, transgender; cis, cisgender. Scale bar: 100μm. H, Hoechst nuclear staining.

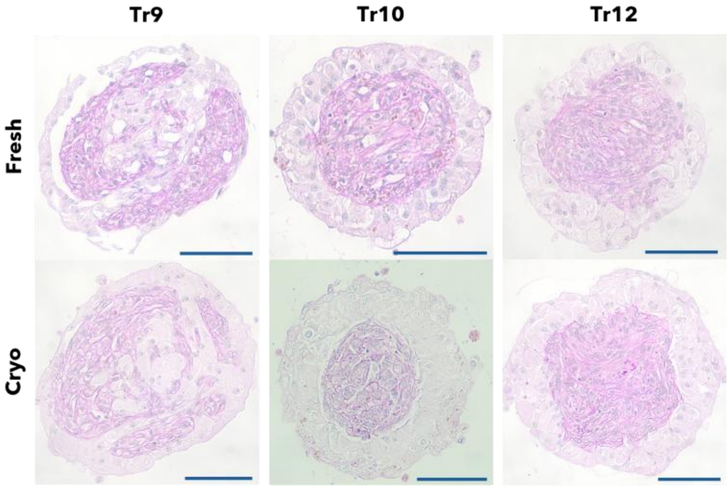

Supplementary Figure S4. Comparison of trans organoids derived from fresh and cryopreserved tissue. H/PAS-stained sections of trans organoids at day 14. Organoids derived from both fresh and cryopreserved (cryo) testicular tissue show comparable histological architecture. Scale bar: 100 μm.

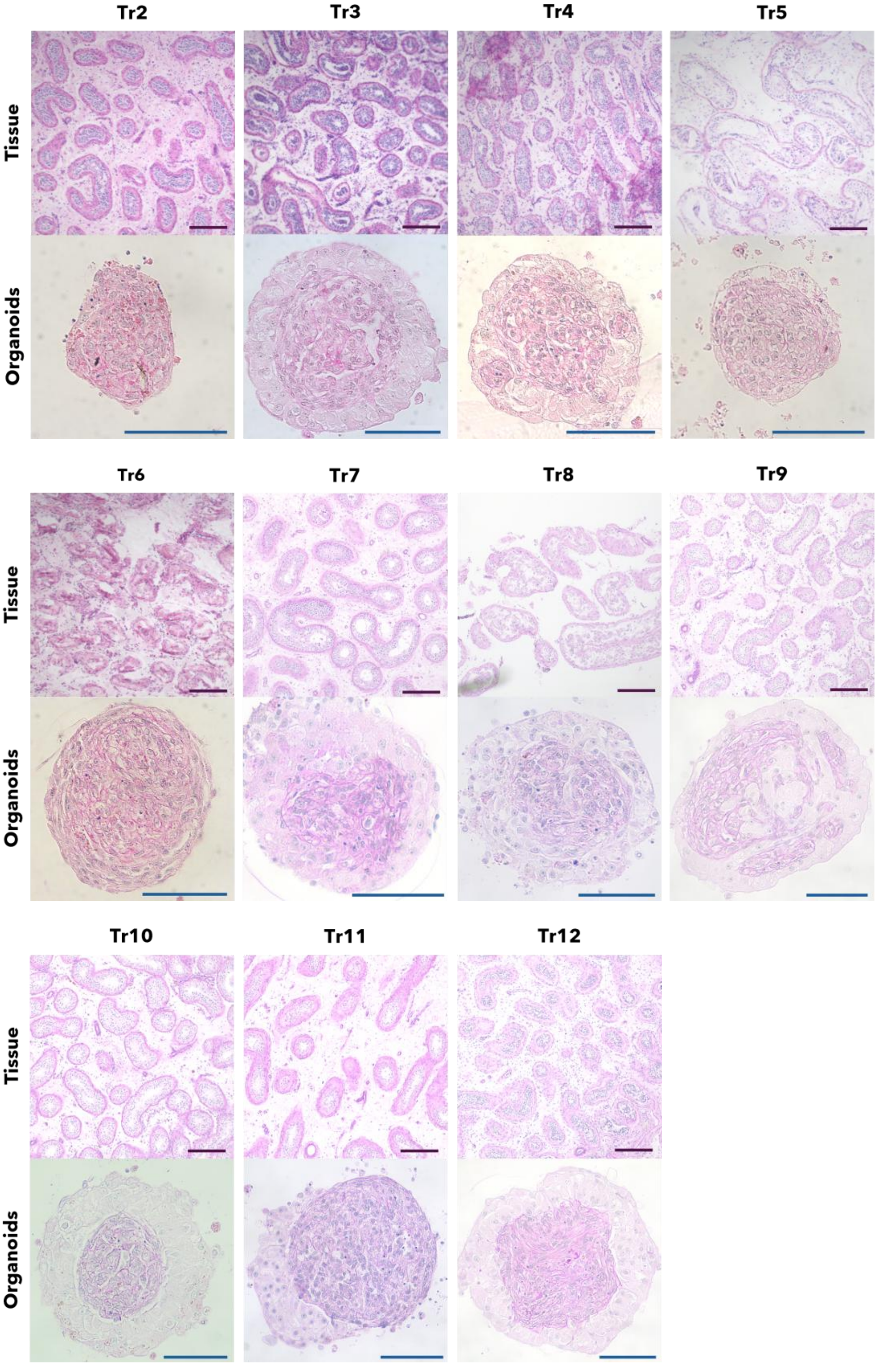

Supplementary Figure S5. Histological comparison of transgender tissue-derived organoids and their tissues of origin at day 14. H/PAS-stained sections show that organoids derived from Tr3, Tr4, Tr7, Tr8, Tr9, Tr10, and Tr12 form bicompartmental structures, while those from Tr2, Tr5, Tr6, and Tr11 do not. Representative images of both organoids and corresponding donor tissues are shown. Purple scale bar: 500 µm; blue scale bar: 100 µm.

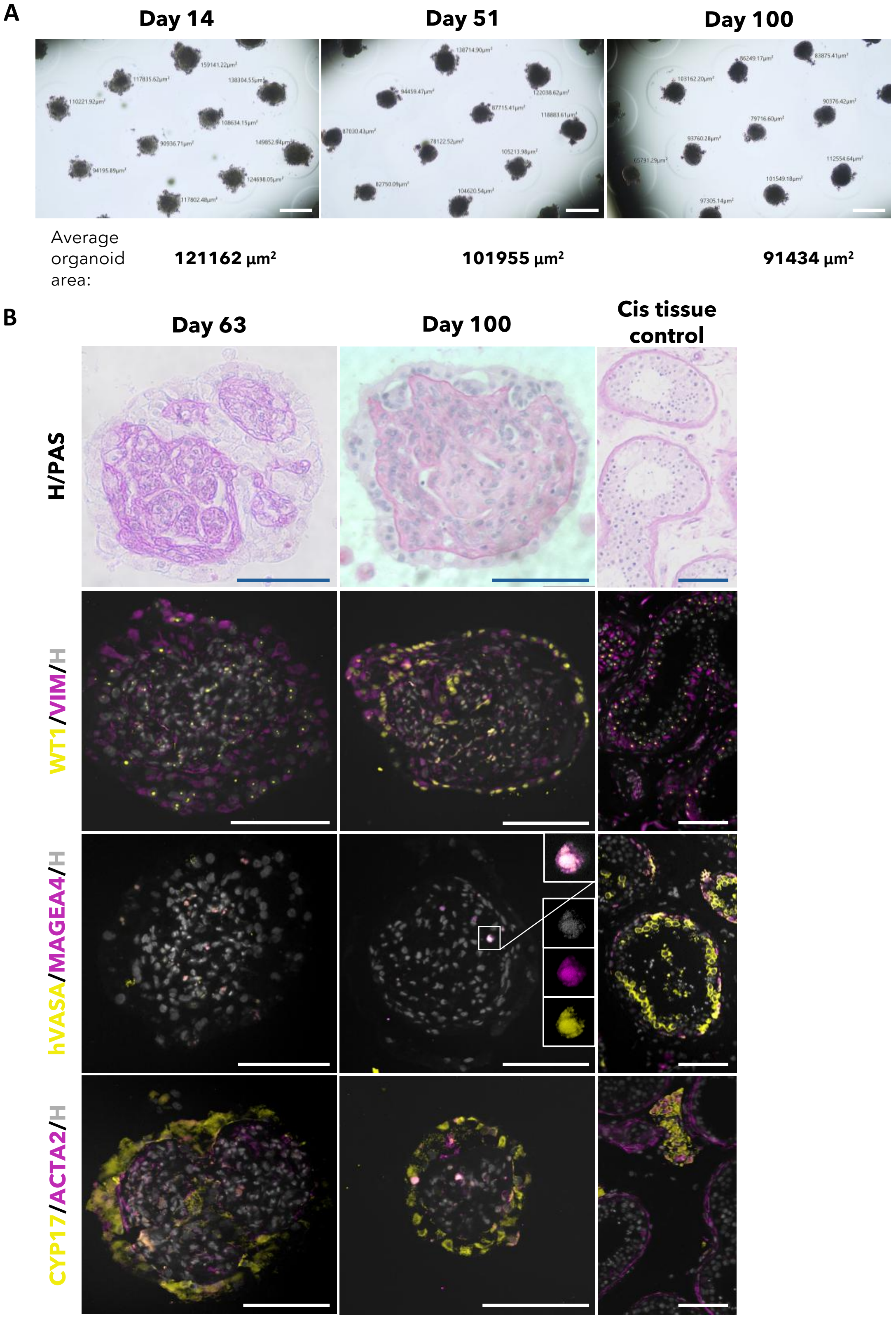

Supplementary Figure S6. Long-term organoid cultures: 63 and 100 days. (**A**) Brightfield images of organoids in 3DPDs show progressive compaction over time, with a significant reduction in average organoid area from day 14 to day 100 (p=0.0018, unpaired two-tailed t-test). Scale bar: 500 µm. (**B**) Long-cultured organoids maintain architecture and present important testicular markers: WT1/VIM for Sertoli cells, hVASA/MAGEA4 for germ cells, CYP17/ ACTA2 for Leydig and peritubular myoid cells. WT1, Wilm's tumour gene 1; VIM, vimentin; hVASA, human VASA; MAGEA4, melanoma-associated antigen 4; CYP17, cytochrome P450 17A1; ACTA2, actin alpha 2. Cis, cisgender. Scale bar: 100 µm. H, Hoechst nuclear staining.

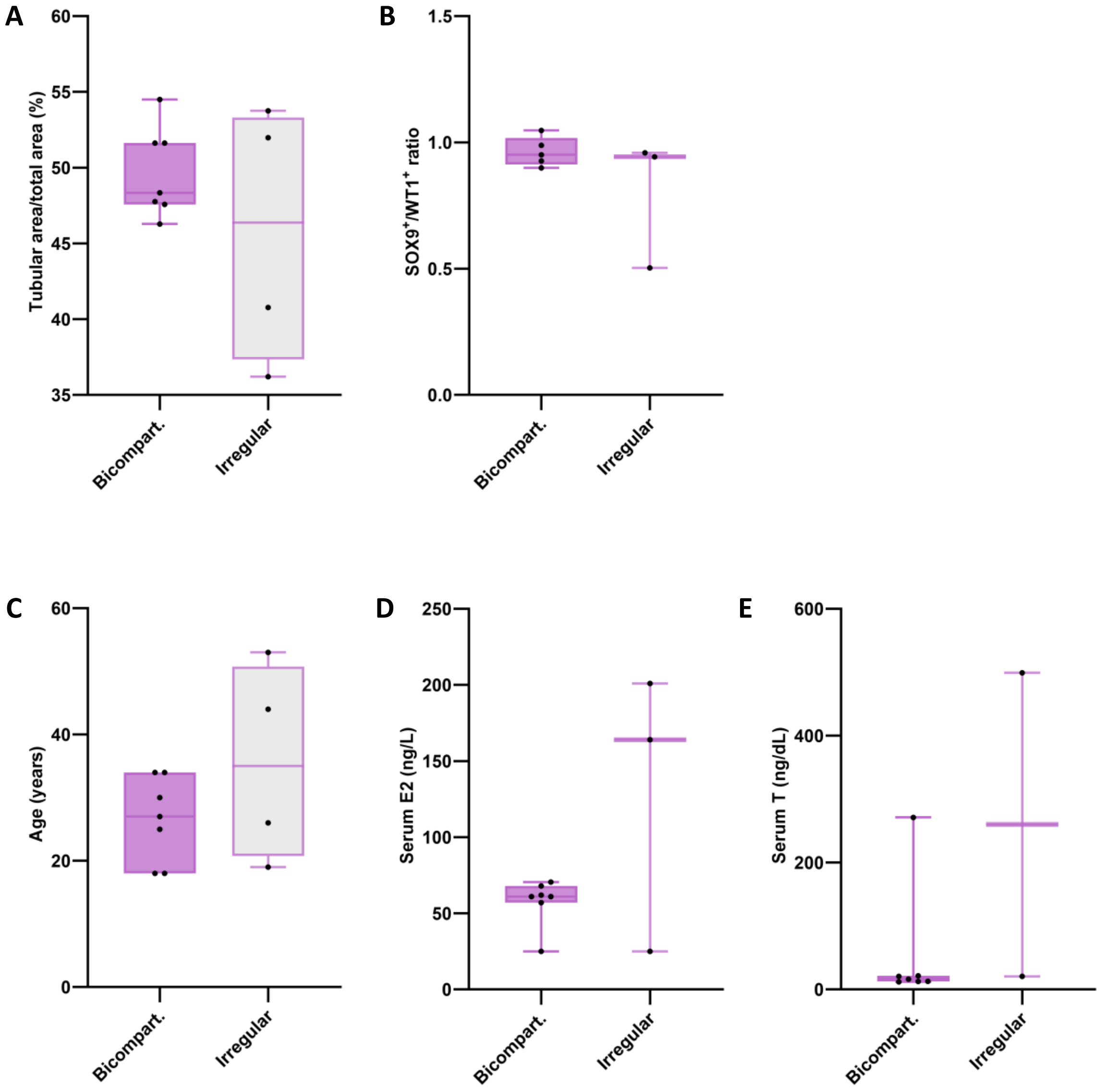

Supplementary Figure S7. "Bicompartmental" vs "irregular" transgender tissues. (A) Percentage of tubular area over total area of the tissue section. **(B)** Sertoli cell assessment for SRY-box transcription factor 9 (SOX9)$^{+}$/Wilm's tumour gene 1 (WT1)$^{+}$ ratio. **(C)** Age at the time of orchidectomy. **(D)** Oestradiol (E2) and **(E)** Testosterone (T) serum levels at the closest analysis to orchidectomy. For statistical evaluation, Mann Whitney test was used. No statistical significances were found.

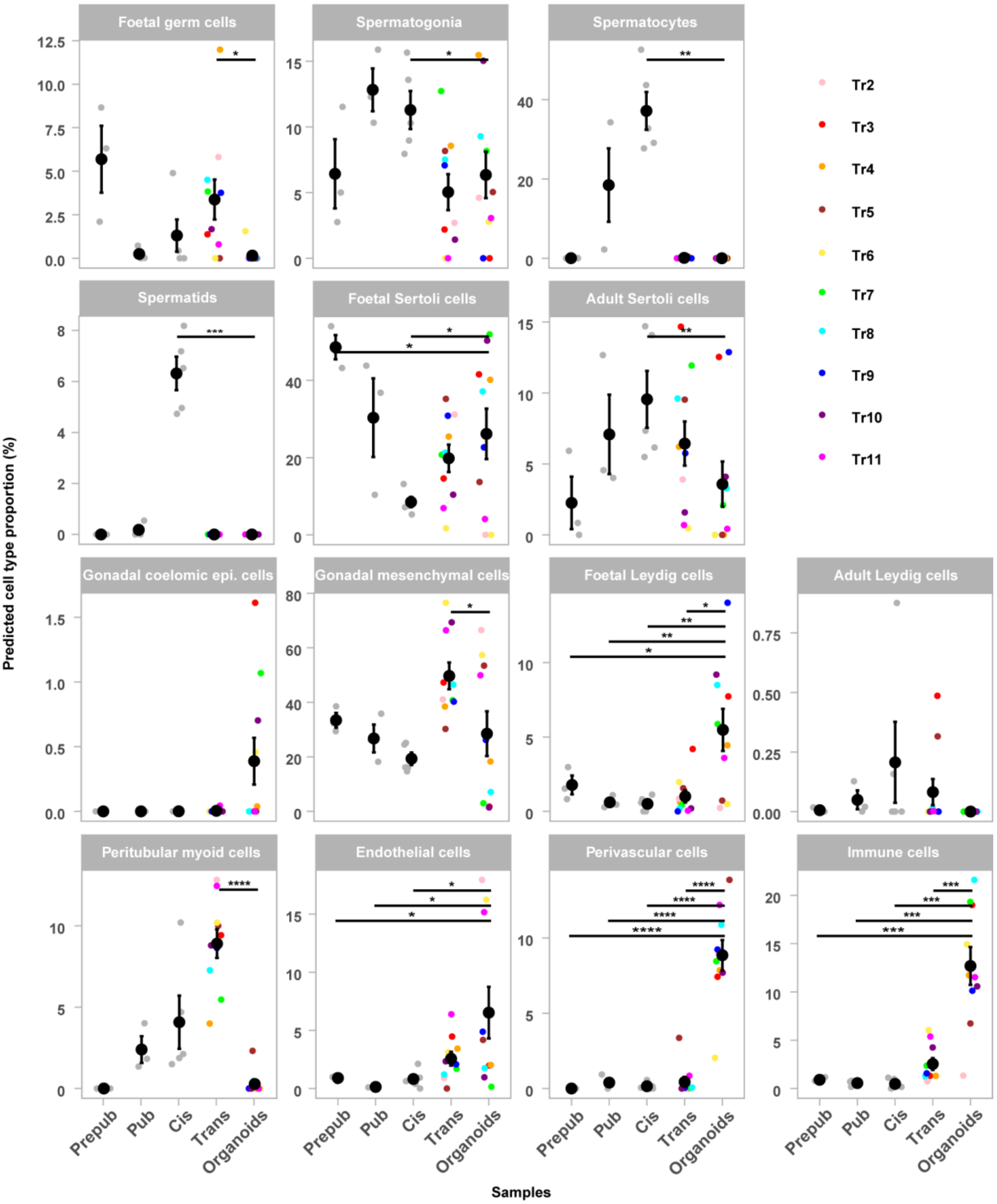

Supplementary Figure S8. Deconvolved bulk RNAseq data showing cell populations and their predicted proportions present in prepub (n=3), pub (n=3), cis (n=5), and trans (n=10) tissues, and trans organoids (n=10). Cell types that were not represented in the samples or did not have any apparent biological relevance are not plotted. Each data point in a graph represents an individual sample. Trans tissue samples and the organoids they generate are colour-coded. For statistical evaluations, the Student's t-test implemented in R was used. *p<0.05; **p<0.01; ***p<0.001; ****p<0.0001.

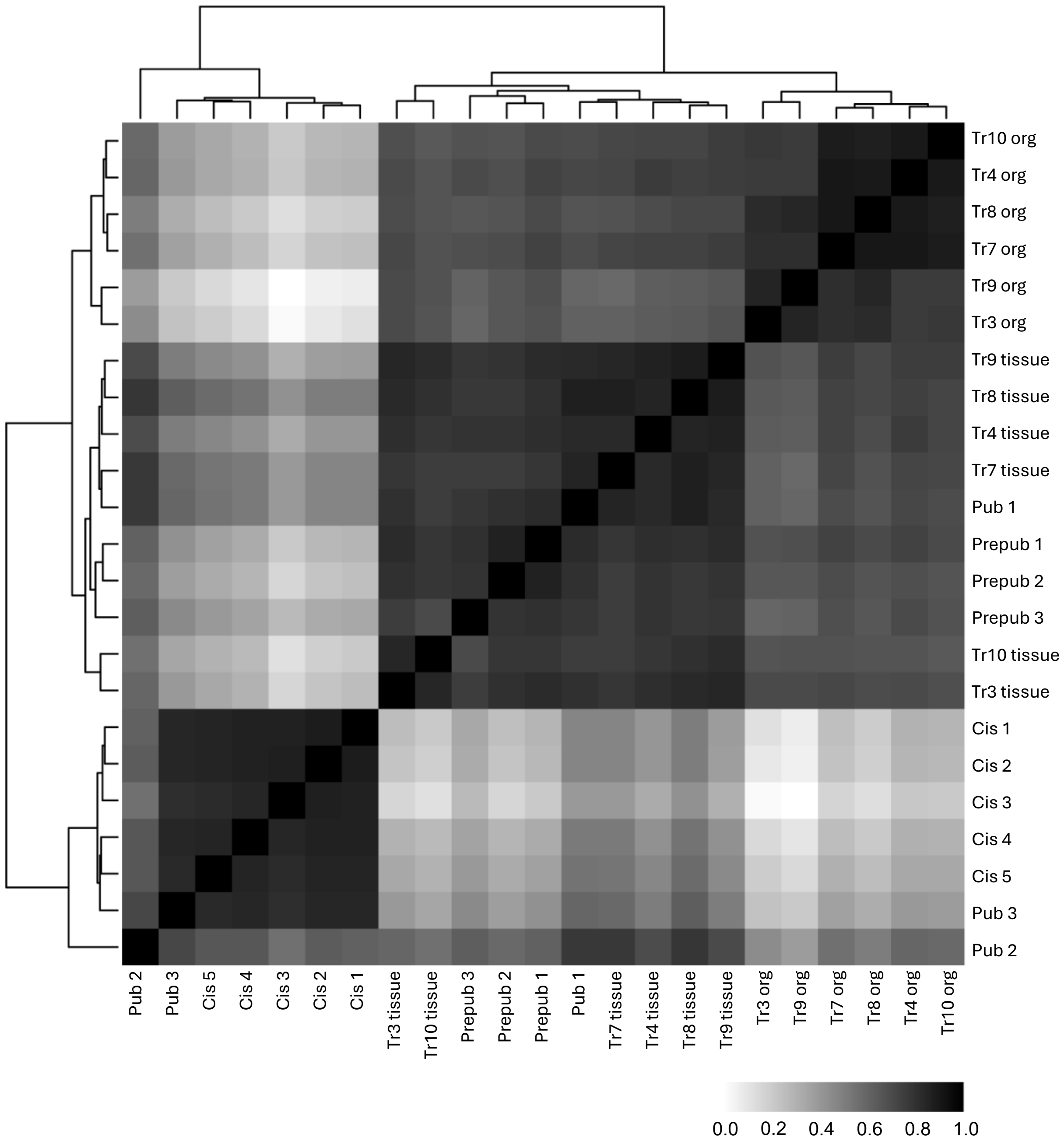

Supplementary Figure S9. Correlation matrix between prepubertal, pubertal, cisgender, and "bicompartmental" transgender (n=6) tissues, and bicompartmental organoids. Pairwise correlation coefficients were determined between gene expression levels across all genes in the RNA-seq dataset. The white to black gradient represents correlation strength, ranging from 0 (no correlation) to 1 (strong positive correlation). Prepub, prepubertal; pub, pubertal; cis, cisgender; tr, transgender; org, organoids.

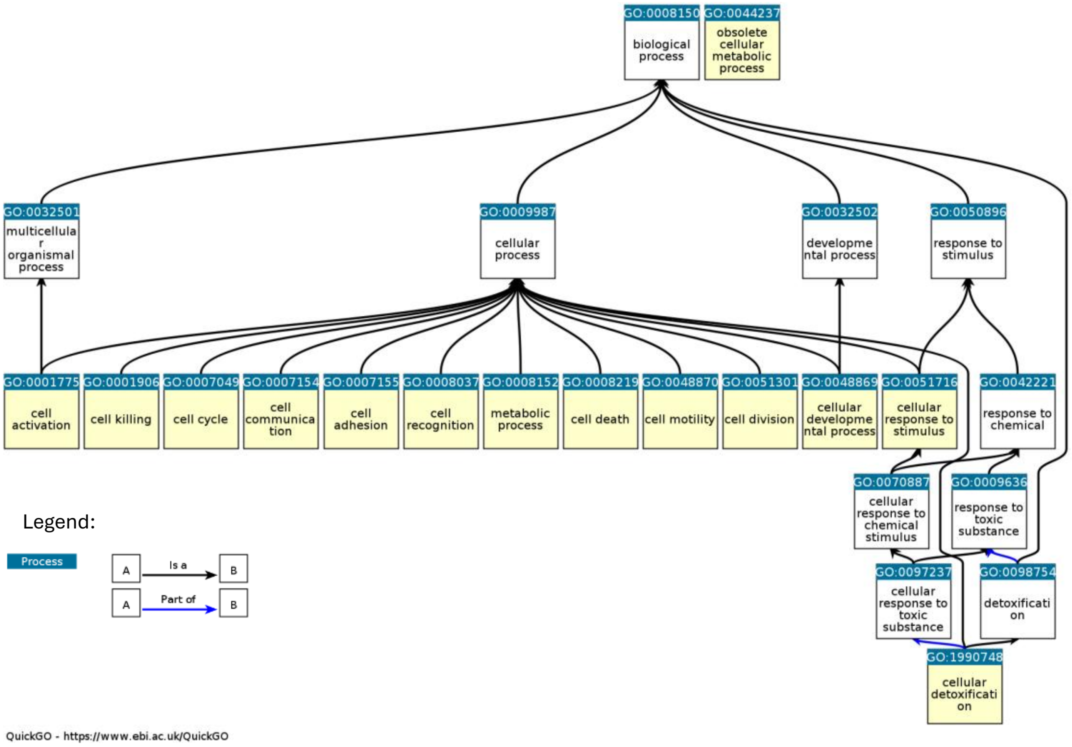

Supplementary Figure S10. Graphical representation of the hierarchical relationships between the GO terms used in the functional analysis. The thirteen selected GO terms are in the yellow boxes. “Cellular metabolic process” (GO: 0044237) shows as obsolete because it is included in the graphic under the name “metabolic process” (GO: 0008152). The graphic was generated at https://www.ebi.ac.uk/QuickGO.
